# Towards precision critical care management of blood pressure in hemorrhagic stroke patients using dynamic linear models

**DOI:** 10.1371/journal.pone.0220283

**Published:** 2019-08-05

**Authors:** Yuzhe Liu, Jody Manners, Yazan Bittar, Sherry H-Y. Chou, Vanathi Gopalakrishnan

**Affiliations:** 1 Department of Biomedical Informatics, University of Pittsburgh, Pittsburgh, PA, United States of America; 2 Department of Neurology, Naval Medical Center Portsmouth, Portsmouth, VA, United States of America; 3 Lewis Katz School of Medicine, Temple University, Philadelphia, PA, United States of America; 4 Department of Critical Care Medicine, University of Pittsburgh School of Medicine, Pittsburgh, PA, United States of America; 5 Department of Neurology, University of Pittsburgh School of Medicine, Pittsburgh, PA, United States of America; 6 Department of Neurosurgery, University of Pittsburgh School of Medicine, Pittsburgh, PA, United States of America; Beth Israel Deaconess Medical Center, UNITED STATES

## Abstract

Finding optimal blood pressure (BP) target and BP treatment after acute ischemic or hemorrhagic strokes is an area of controversy and a significant unmet need in the critical care of stroke victims. Numerous large prospective clinical trials have been done to address this question but have generated neutral or conflicting results. One major limitation that may have contributed to so many neutral or conflicting clinical trial results is the “one-size fit all” approach to BP targets, while the optimal BP target likely varies between individuals. We address this problem with the Acute Intervention Model of Blood Pressure (AIM-BP) framework: an individualized, human interpretable model of BP and its control in the acute care setting. The framework consists of two components: one, a model of BP homeostasis and the various effects that perturb it; and two, a parameter estimator that can learn clinically important model parameters on a patient by patient basis. By estimating the parameters of the AIM-BP model for a given patient, the effectiveness of antihypertensive medication can be quantified separately from the patient’s spontaneous BP trends. We hypothesize that the AIM-BP is a sufficient framework for estimating parameters of a homeostasis perturbation model of a stroke patient’s BP time course and the AIM-BP parameter estimator can do so as accurately and consistently as a state-of-the-art maximum likelihood estimation method. We demonstrate that this is the case in a proof of concept of the AIM-BP framework, using simulated clinical scenarios modeled on stroke patients from real world intensive care datasets.

## Introduction

Balancing the need for cerebral perfusion and the risk of hemorrhagic conversion or rehemorrhage make post-stroke blood pressure (BP) control a challenging problem. [1, 2] Proper BP management in the intensive care unit (ICU) following a stroke remains an unresolved issue, despite numerous trials studying the effect of antihypertensives in stroke care. Both ischemic and hemorrhagic stroke patients often exhibit elevated BP during initial treatment, but it is as yet unclear whether lowering BP results in worse, better, or no difference in outcomes. [1–5] One study found a U-shaped effect of BP on outcomes. [6] In addition, studies on raising BP in the treatment of ischemic stroke have found no difference in the rate of complications. [7] The inconsistency of these results are perhaps not surprising when we consider that each individual stroke patient functioned at different baseline BPs before their stroke and had different degrees and areas of brain injury during their stroke. Thus, a monolithic BP target for all stroke patients, as seen in many of these trials, may be beneficial for some but detrimental for others. We hypothesize that individualized BP goals and treatment regimens will produce better outcomes than the current one-size-fits-all approach.

Current American Heart Association (AHA) ischemic stroke management guidelines, updated in 2013 with minor updates in 2018, recommend antihypertensives in patients when BP is greater than 220/120 mmHg and not receiving thrombolytics, or greater than 185/110 mmHg in patients who receive thrombolytics. [4, 8] These guidelines have not changed since the previous edition in 2007. [9] Current studies examining the effect of BP control post-stroke include the ENCHANTED trial, [10, 11] where the BP arm of the trial is scheduled to be completed in 2018. Current AHA guidelines for primary intracerebral hemorrhage (ICH) state acute BP lowering to 140 mmHg systolic is safe in patients with systolic pressures of 150-220 mmHg, and that aggressive BP lowering may be considered, even if its safety is unknown, in patients whose systolic pressures are even higher. [12] These guidelines provide no BP target for ischemic stroke patients and one SBP target for every patient with primary ICH. We believe that individualized BP targets will improve clinical outcomes.

The question of aggressive vs conservative BP lowering, as suggested in the primary ICH guidelines, currently rely on clinical experience or institutional best practice guidelines to decide which drug will be most effective for whom. In practice, this often results in a couple of equally valid choices for management, with the specifics left to a physician’s practice style. This in turn, often depends on a physician’s experiences and observations about the effectiveness of medications she’s used to lower BP. These observations, however, are confounded by many factors, such as non-linear pharmacodynamics, inaccurate measurements of BP, natural deviations of BP, uncertainty regarding timing of medication administration and observed effects, usage of multiple drugs in the same patient, biases in the patient population each individual physician sees, and cognitive biases in remembering past results. In order to study which medications are more effective in which people, an objective, quantitative approach is needed. Ideally, this approach should be able to use data from existing medical records.

### Quantitative approach to calculating drug effectiveness

**Fig 1:**
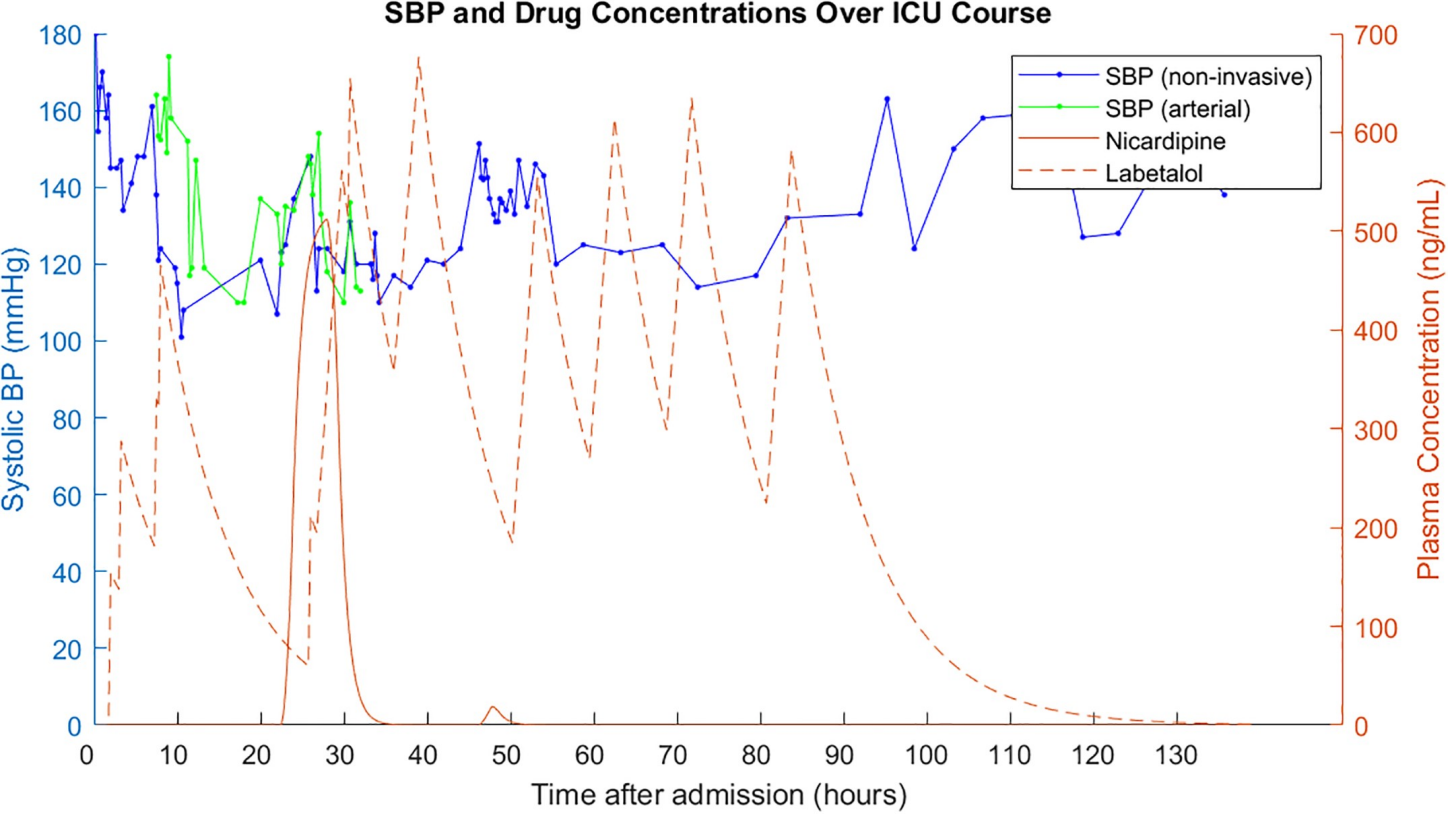
Systolic BP and concentrations of two BP meds over an ICU course. BP data and medication admins taken from an ICH patient in an ICU dataset from UPMC. Systolic BP can be measured using both non-invasive methods and an arterial line. Drug concentrations are estimated from the medication administration record based on drug pharmacokinetics data.

Suppose that we have BP data from the medical record of a stroke patient during his stay in the ICU. Suppose also that we can estimate concentrations of antihypertensives from the medication administration record using pharmacokinetics data. We have such an example from an ICH dataset extracted from the University of Pittsburgh Medical Center (UPMC) Presbyterian Hospital medical records (IRB PRO16110384). In order to study the effects of a drug on systolic BP, we might be able to plot a curve like Fig 1.

Just from glancing at the data, it is difficult to judge whether labetalol or nicardipine is more effective at lowering systolic BP. First, we have to define what effectiveness is: Is it the amount of mmHg drop per drug concentration? Second, we see that labetalol has many peaks, each of which may be associated with different amounts of SBP drop. Third, there are periods of time during which the arterial line reading differs from the non-invasive reading. Which one should we trust, and should we correct the other one? Fourth, we see that measurements of BP are variable. How much is measurement noise, how much is actual BP variations, and how much is from the effects of medication? These are all reasons why a qualitative assessment by a physician may be error-prone. A quantitative assessment will be more objective. Fortunately, the problem we are describing here is equivalent to calculating dose-response relationships in the field of pharmacodynamics.

**Fig 2:**
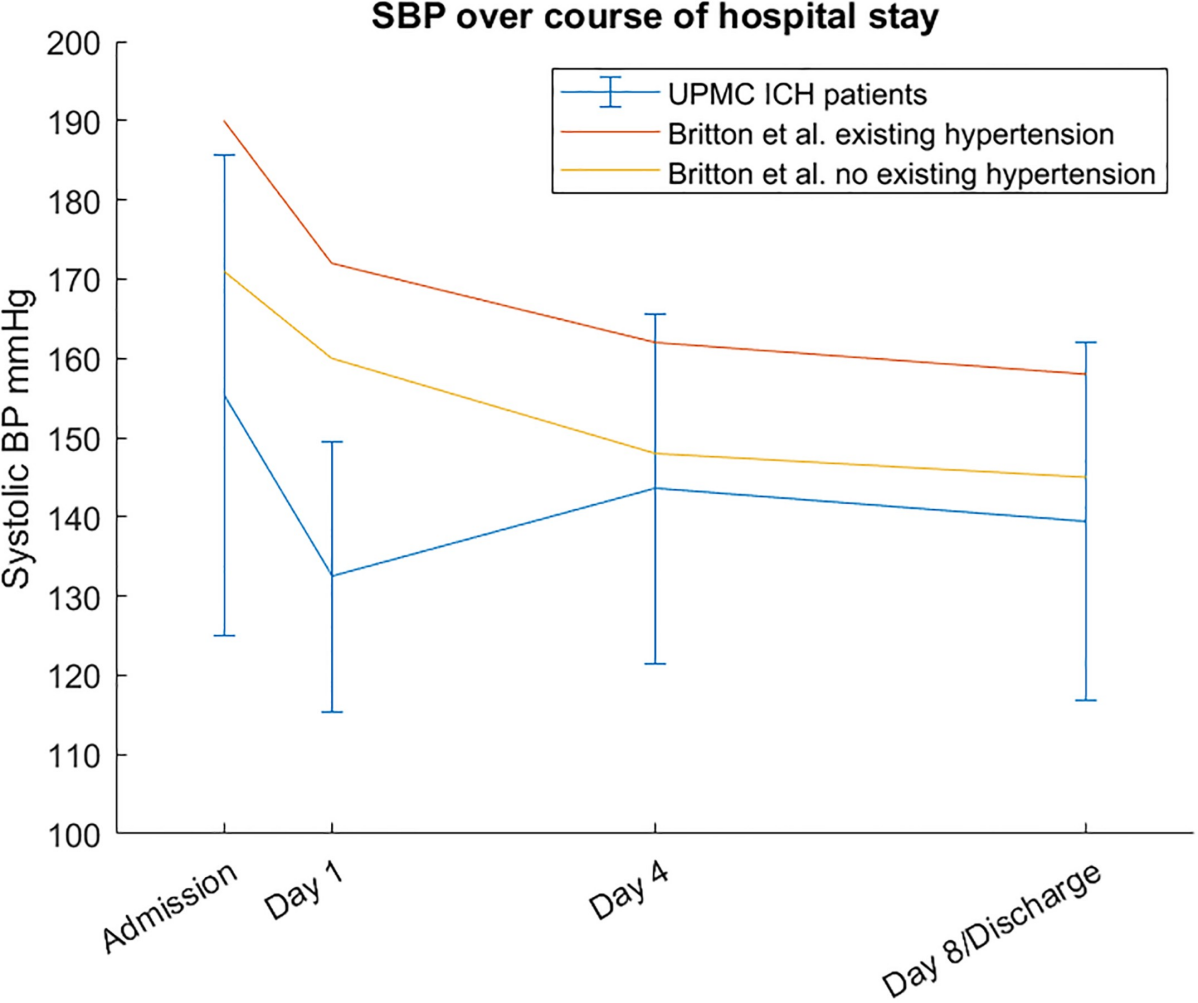
Spontaneous systolic BP for UPMC intracerebral hemorrhage compared to Britton et al. results. Mean SBP for UPMC ICH patients compared with Britton et al.’s results from stroke patients with and without existing hypertension. Error bars for UPMC data denote 1 standard deviation. For the last data point, we provided SBP on day 8, while Britton et al. only reported SBP data for day of discharge.

The *E*_*max*_ model is a popular model for studying dose-response relationships, and is usually fit using maximum likelihood estimation via a non-linear regression method such as Gauss-Newton or Levenberg-Marquardt. [13–15] Most studies of drug dose vs response, however, are conducted in a tightly controlled experimental setting, where the drug effect is assumed to be the only effect. Four our ICH patient, this is not the case; there is no guarantee that his systolic BP would have remained constant in the absence of his BP meds. In fact, all patients, and particularly patients with both hemorrhagic and ischemic stroke, typically have elevated BP at the beginning of their hospital course that then decreases spontaneously over the course of their stay without administration of new antihypertensive drugs. [16–19] Britton et al. studied spontaneous BPs for stroke patients and controls, and their systolic BP results are compared to those from the UPMC ICH dataset in Fig 2. [17] In contrast to the Britton et al. results, the UPMC dataset is only with primary intracerebral hemorrhage patients, while Britton et al. included both hemorrhagic and ischemic stroke. In addition, the UPMC dataset consists of patients in which antihypertensives were used to rapidly achieve a goal target of less than 140 mmHg SBP, while Britton et al. studied patients in which no additional antihypertensives were used.

We can see that in general, there appears to be a positive perturbation to BP apparent during admission that eventually decreases back to a homeostatic baseline in a manner similar to exponential decay. By combining a model of this perturbation of BP homeostasis with an *E*_*max*_ model of drug effectiveness, we can more accurately study the effectiveness of BP meds in the setting of stroke.

### A quantitative model for the study of BP control in stroke

We propose a model of BP dynamics which assumes that BP hovers around a homeostatic level unless otherwise perturbed, such as in acute stroke. We propose a model of BP homeostasis perturbations in acute stroke patients that consists of two chief components: One the natural time trend of BP over time, and two, the effect on BP from medications. We model the natural time trend as an exponentially decaying curve, as seen in patients in Britton et al. [17] We model the effect of medications using an *E*_*max*_ model, also known as a Hill equation or Michaelis-Menten equation model. [14]

We design a framework that we call the Acute Intervention Model of Blood Pressure (AIM-BP). AIM-BP is a first order discrete dynamic linear model (DLM) that models the body’s natural tendency towards homeostasis, the effect of external interventions on BP and heart rate (HR), and the noisiness inherent in the system as well as in their measurement. By modeling these separate effects together, we hope to better separate the various effectors of a patient’s BP in the ICU in order to facilitate clinical research on the effect of critical care BP management of stroke.

A major limiting factor on this kind of clinical research is the quality of clinical data available. Although high frequency measurements of BP can be done in the ICU, realistically most recorded clinical data only has charted BP data to work with. While charted data can be from multiple modalities (cuff and arterial line), they are usually recorded at irregular intervals ranging from minutes to an hour, sometimes with longer periods of time where measurements are missing. DLMs provide an easily extensible framework that intuitively handles missing data and multi-modal measurements, two critical features when working with frequently measured vital sign data. DLMs are a popular class of time series models, and have been used to predict post-surgical blood count values, [20, 21] perform outbreak surveillance, [22] and estimate values in a noisy setting in a wide variety of medical and non-medical problems. Intuitively, DLMs calculate the best estimate of a true parameter by combining information about the parameter from the previous time point and noisy measurements of the parameter at the current time point. We refer the reader to an introductory textbook on DLMs for a more in depth explanation. [23]

BP modeling in the critical care setting has been studied mostly in the context of high resolution, beat-to-beat tracking. [24–26] The Kalman filter (e.g. dynamic linear models) has been used for modeling BP in this context for the purposes of reducing artifacts. [25] Lehman et al. modeled ICU BP and HR data using switching vector autoregressive models (SVARs). [26–28] SVARs are a form of autoregressive model very similar to DLMs, with the removal of a hidden layer and the inclusion of a set of dynamic modes through which a time series can switch during its course—in effect, SVARs model time series as a mixture of DLMs without the hidden layer. Lehman et al. have applied this model to data from the Medical Information Mart for Intensive Care (MIMIC) database, a publicly available dataset of electronic health record data from critical care units at Beth Israel Deaconess Medical Center, [29], to predict mortality and analyze BP variability. The advantage to the SVAR-based method used by Lehman et al. is its ability to learn dynamic modes applicable across a whole population of patients and weight them separately for each patient.

There are several disadvantages to using SVAR for our purposes, however. While the dynamic modes learned by SVAR are useful for classification, they lack human interpretability. In addition, the inclusion of multiple dynamic modes with multiple transition matrices, in addition to the weights for each dynamic mode at each time point for each patient, add up to much more parameters to estimate compared to a single DLM. Lehman et al. accomplish this by using expectation maximization on high density data from MIMIC, using the MIMIC waveform database that contains beat to beat BP information with low amounts of missing data. [27]

For the purposes of identifying BP trends in stroke management in the ICU, we are interested in longer term, lower resolution measurements of BP, with a time scale on the order of minutes, hours, and even days. Studies at these time scales typically use more rudimentary models of BP, such as mean, least squares slope, and standard deviation of the mean arterial pressure. [30]

After our review of the literature, we decided that a clinically useful model of BP management for stroke in the ICU setting must satisfy the following requirements:

1. Model multivariate time series data of BP and HR at a minute to hour timescale. This data will include irregularly measured or missing values, as well as measurements of the same variable via multiple modalities with their various measurement noises.
2. Provide human-interpretable, clinically relevant model parameters.
3. Model body homeostasis mechanisms and disease state perturbations to homeostasis, such as the acute increase followed by spontaneous decrease of BP often found in stroke patients.
4. Model the effects of ICU interventions, such as BP medication.

Currently, no model exists that satisfies all of these requirements. We are particularly interested in separating out requirements 3 and 4. Separating out the effects of drugs versus a patient’s natural BP time course is necessary in the context of several research questions: One, which drugs are more effective for which people in the management of BP after a stroke? Two, can we better determine drug effectiveness during acute care compared to current clinician judgment? Three, does the relationship between managed BP and the natural homeostasis level correlate with outcomes? To our knowledge, we are the first to design a modeling framework that can satisfy the above requirements and answer these research questions.

**Fig 3:**
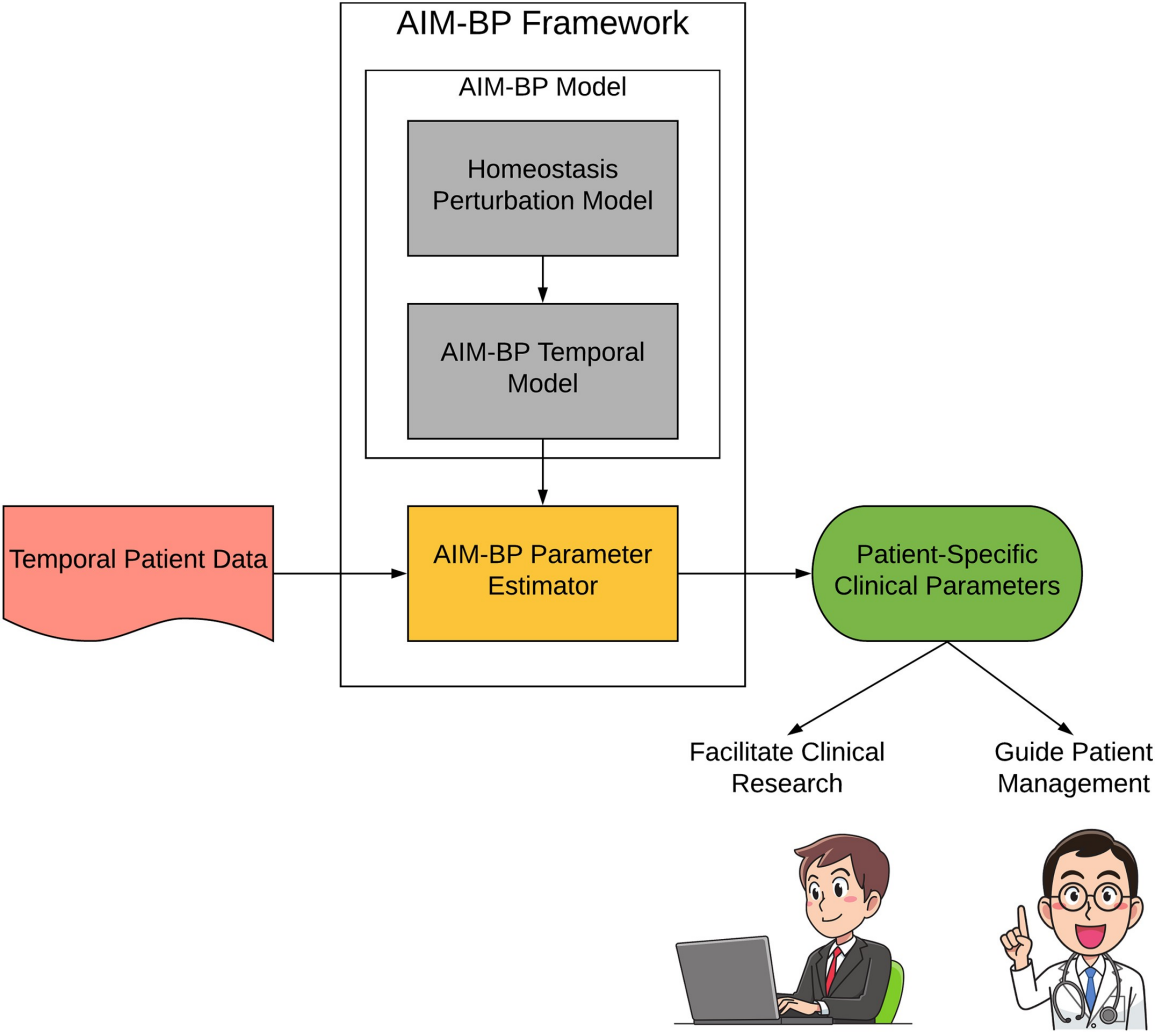
AIM-BP framework. The AIM-BP framework consists of two parts: The model specification (a structured dynamic linear model) and the parameter estimator (a MCMC-based sampler).

The AIM-BP framework consists of two main parts (Fig 3). The first is the AIM-BP model specification, which is composed of a homeostasis perturbation model and a DLM-based AIM-BP temporal model. The second is the AIM-BP parameter estimator, which takes data and learns parameters of an AIM-BP model instance using Markov Chain Monte Carlo (MCMC) sampling. MCMC methods have gained popularity over methods like maximum likelihood estimation and expectation maximization due to their flexibility in modeling non-Gaussian probabilities and non-linear extensions to DLMs. [31–33]

We hypothesize that the AIM-BP framework will identify parameters that describe a patient’s spontaneous trends and drug pharmacodynamics at least as accurately and consistently as a current state-of-the-art maximum likelihood estimation method.

## Materials and methods

The work included research on human subjects approved by the University of Pittsburgh Institutional Review Board (IRB PRO16110384). Consent for data usage was obtained in written form. Data was analyzed anonymously.

### Model design

The AIM-BP model consists of two components: A homeostasis perturbation model of BP behavior in the acute stroke setting and a DLM-based AIM-BP temporal model. The homeostasis perturbation model is a general model of BP behavior that can be used independently of the AIM-BP temporal model. We describe both components of the AIM-BP model here.

### Homeostasis perturbation model

The homeostasis perturbation model combines an exponential decay model (*P*_*spon*_(*t*)) of the natural time course of BP in an ICU stroke patient and an *E*_*max*_ model (*P*_*med*_(*t*)) of antihypertensive drug effects. It can be described as follows:

$$\begin{array}{c} P_{spon}\left(t\right)=B_{max}*{\left(1-r_{B}\right)}^{t} \end{array}$$

$$\begin{array}{c} P_{med}\left(t\right)=\frac{E_{max}*c\left(t\right)}{EC_{50}+c\left(t\right)} \end{array}$$

$$\begin{array}{c} SBP(t)=P_{spon}\left(t\right)+\sum_{meds}P_{med}\left(t\right)+SBP_{H}+\epsilon_{S} \end{array}$$ (1)

$$\begin{array}{c} DBP(t)=ratio_{SD}*\left(P_{spon}\left(t\right)+\sum_{meds}P_{med}\left(t\right)\right)+DBP_{H}+\epsilon_{D} \end{array}$$ (2)

Where *B*_*max*_ and *r*_*B*_ parameterize the exponential decay of the spontaneous change in acute stroke patients, and *E*_*max*_ and *EC*_50_ for each drug parameterize the *E*_*max*_ model for each drug, *c*(*t*) is the drug concentration at time *t*. *SBP*_*H*_ and *DBP*_*H*_ are homeostasis levels of systolic BP (SBP) and diastolic BP (DBP) without the effects of the perturbations. *ratio*_*SD*_ describes the proportional effect of the perturbation on diastolic pressure compared to the effect on systolic pressure. *ϵ*_*S*_ and *ϵ*_*D*_ are normal white noise terms.

In our stroke homeostasis perturbation model, we model two types of perturbations. The first is the increased BP post stroke, which often decreases spontaneously throughout the ICU stay. We model this perturbation as an initial perturbation that decays exponentially to zero, such that the BP eventually converges to a stable homeostasis value *SBP*_*H*_. In our model, we allow both positive and negative perturbations, such that if a patient initially had a drop in BP that then recovered (e.g. in the setting of shock), we could model that as well. Furthermore, by varying the rate at which BP converges to *SBP*_*H*_, we can model quick returns to homeostasis vs a more steady, almost constant return. What this model does not capture are situations in which a constant homeostatic BP is never achieved. As such, cyclic effects such as the sleep-wake effect on BP are not captured. Instead, these effects will factor into the noise components of the model.

The second class of perturbations modeled are the effects of medications used in the management of the patient’s cardiovascular state, with a separate *P*_*med*_(*t*) for each medication used. We chose the *E*_*max*_ model of pharmacodynamics to model these medications. Put together, the homeostasis perturbation model combines an exponential decay model of spontaneous behavior in acute stroke (*P*_*spon*_(*t*)) in addition to *E*_*max*_ models of drug pharmacodynamics (each *P*_*med*_(*t*)). This homeostasis perturbation model is a general model of BP behavior, and can be fit to data without using the AIM-BP framework.

### Dynamic linear model

We describe the dynamic linear model that underlies the AIM-BP framework. This system will be tested on simulated data to demonstrate the feasibility of the framework. This model contains parameters that will be held constant for the purposes of this paper to simplify evaluation, but can be learned in the future when more data is available for a richer description of BP control. The AIM-BP dynamic linear model is characterized by the following variables:

#### Observed variable: y

The variable *y* = {*y*_1_, *y*_2_, …*y*_*T*_} describes observed measurements of BP and HR. At a given time point *t*, *y*_*t*_ contains all measurements of systolic blood pressure (SBP), diastolic blood pressure (DBP), and heart rate (HR). For example, we could have measurements from an automated pressure cuff and an arterial line, and a HR measurement from an O2 saturation monitor. Our *y* variable space would then look like this:

$$\begin{array}{c} y=[\begin{array}{c} y^{\left(1\right)}\\ y^{\left(2\right)}\\ y^{\left(3\right)}\\ y^{\left(4\right)}\\ y^{\left(5\right)} \end{array}]=[\begin{array}{c} \text{Cuff}\;\text{measured}\;\text{SBP}\\ \text{Cuff}\;\text{measured}\;\text{DBP}\\ \text{Arterial}\;\text{line}\;\text{measured}\;\text{SBP}\\ \text{Arterial}\;\text{line}\;\text{measured}\;\text{DBP}\\ \text{O2}\;\text{saturation}\;\text{monitor}\;\text{measured}\;\text{HR} \end{array}] \end{array}$$

By capturing all measurements of a vital sign at a given time point, our model can better estimate the true value of these vital signs. For the purposes of the simulations in this paper, however, we will use a simplified observed measurement space as follows:

$$\begin{array}{c} y=[\begin{array}{c} y^{\left(1\right)}\\ y^{\left(2\right)}\\ y^{\left(3\right)} \end{array}]=[\begin{array}{c} \text{Cuff}\;\text{measured}\;\text{SBP}\\ \text{Cuff}\;\text{measured}\;\text{DBP}\\ \text{O2}\;\text{saturation}\;\text{monitor}\;\text{measured}\;\text{HR} \end{array}] \end{array}$$

#### Hidden variable: x

We design a hidden variable *x* = {*x*_1_, *x*_2_, …*x*_*T*_} that describes the true underlying BP and HR measurements of a patient. At a given time point *t*, *x*_*t*_ contains information about a patient’s true SBP, DBP, and HR, as well the baseline homeostasis target for SBP, DBP, and HR. The homeostasis target for SBP, DBP, and HR models what the body’s natural desired BP is absent outside interventions. Our AIM-BP model will model the tendency of these vital signs to trend towards a homeostatic value. Finally, we include a constant value in *x* to allow for the expression of non-white noise.

$$\begin{array}{c} x=[\begin{array}{c} x^{\left(1\right)}\\ x^{\left(2\right)}\\ x^{\left(3\right)}\\ x^{\left(4\right)}\\ x^{\left(5\right)}\\ x^{\left(6\right)}\\ x^{\left(7\right)} \end{array}]=[\begin{array}{c} \text{SBP}\\ \text{DBP}\\ \text{SBP}\;\text{homeostasis}\;\text{target}\\ \text{DBP}\;\text{homeostasis}\;\text{target}\\ \text{HR}\\ \text{HR}\;\text{homeostasis}\;\text{target}\\ \text{Constant} \end{array}] \end{array}$$

#### Input variable: u

The input variable *u* = {*u*_1_, *u*_2_, …*u*_*T*_} represents all the various perturbations of the BP and HR system from the homeostasis perturbation model described previously. For the sake of simplicity in the following simulations, this proof of concept will model two major modes of drug delivery for acute hypertension treatment in ICU settings: Intravenous (IV) labetalol and IV nicardipine. Labetalol, a beta-adrenergic blocking agent, is modeled here as an IV bolus push medication to immediately lower BP. Nicardipine, a calcium channel blocker, is typically given as a continuous IV drip to maintain a target SBP, with titrations of the drip rate as necessary to achieve this. Together, these two medications form an acute treatment regimen for SBP. We do not explicitly model non-linear drug interactions. At a given time point *t*, *u*_*t*_ contains the post-stroke BP perturbation as well as the effect of each drug on the patient. For example, a simple model with two drugs might have an input *u* as:

$$\begin{array}{c} u=[\begin{array}{c} u^{\left(1\right)}\\ u^{\left(2\right)}\\ u^{\left(3\right)} \end{array}]=[\begin{array}{c} \text{Stroke}\;\text{BP}\;\text{perturbation}(P_{spon})\\ \text{IV}\;\text{labetalol}\;\text{bolus}\;\text{effect}(P_{lab})\\ \text{IV}\;\text{nicardipine}\;\text{drip}\;\text{effect}(P_{nic}) \end{array}] \end{array}$$

Translating the stroke perturbation *P*_*spon*_(*t*) to *u*_*t*_:

$$\begin{array}{c} u_{t}^{\left(1\right)}=B_{max}*{\left(1-r_{B}\right)}^{t} \end{array}$$

*B*_*max*_ and *r*_*B*_ are the parameters to estimate.

The *E*_*max*_ model is translated from *P*_*med*_(*t*) to *u*_*t*_ as follows:

$$\begin{array}{c} u_{t}^{\left(2\right)}=\frac{E_{max}^{\left(2\right)}c\left(t\right)}{EC_{50}^{\left(2\right)}+c\left(t\right)} \end{array}$$

Where *c*(*t*) is the drug plasma concentration at time *t*. The same equation applies for $u_{t}^{\left(3\right)}$. In general, each antihypertensive drug used during the ICU course for one patient will have its own *E*_*max*_ and *EC*_50_ parameter that can be used to calculate *u*.

#### Model parameters

The previous variables interact in a dynamic linear model via the following set of equations:

$$\begin{array}{cc} x_{0} & ∼N(\mu_{0},\Sigma_{0})\\ x_{t} & =\Phi x_{t-1}+ϒu_{t}+w_{t}\\ y_{t} & =Ax_{t}+v_{t}\\ w & ∼N(0,Q)\\ v & ∼N(0,R) \end{array}$$

*x*_0_ is the initial value of *x* with mean *μ*_0_ and covariance Σ_0_. Φ is the transition matrix between hidden states from one time point to the next. ϒ is the response matrix to input interventions *u*. *A* is the emission matrix that translates the hidden state *x* to the observed measurements *y*. Finally, *w* and *v* are white noise terms with mean 0 and covariance matrices *Q* and *R*, respectively. Note the presence of a constant term *x*^(7)^ in *x* allows us to model non-zero mean Gaussian measurement noise with a white Gaussian *v*. The covariance of *w* and *v* was set to values close to variances seen in actual data from MIMIC.

Because of the design of our homeostasis target variable, the homeostasis targets (*X*^(3)^ for SBP, *X*^(4)^ for DBP, and *X*^(6)^ for HR) depend solely on initial parameters *μ*_0_ and Σ_0_. We simplify the model by zeroing out the rows and columns of Σ_0_ that correspond to the homeostasis target variables and constants. Thus, the homeostasis targets depend solely on *μ*_0_.

The AIM-BP DLM has a specific structure for Φ and ϒ. These two parameters characterize how a given individual’s vital signs trend over time, as well as how they respond to treatment.

The parameter Φ models the evolution of intrinsic vital signs. It has a specific structure:

$$\begin{array}{c} \Phi =[\begin{array}{ccccccc} 1-r_{h} & 0 & r_{h} & 0 & 0 & 0 & 0\\ 0 & 1-r_{h} & 0 & r_{h} & 0 & 0 & 0\\ 0 & 0 & 1 & 0 & 0 & 0 & 0\\ 0 & 0 & 0 & 1 & 0 & 0 & 0\\ 0 & 0 & 0 & 0 & 1-r_{h} & r_{h} & 0\\ 0 & 0 & 0 & 0 & 0 & 1 & 0\\ 0 & 0 & 0 & 0 & 0 & 0 & 1 \end{array}] \end{array}$$

In this structure, *r*_*h*_ ∈ [0, 1] is a rate value that models various homeostatic mechanisms affecting autonomic regulation of BP and HR, such as vasoconstriction/vasodilation, the renin-angiotensin system, and the baroreceptor reflex. Since the baroreflex operates at a time scale of seconds [34] and has a large effect on short term BP homeostasis, we use a high *r*_*h*_ of 0.9 to model this fast homeostasis mechanism. Although we could theoretically sample and estimate a value for *r*_*h*_, for all practical purposes with the amount of noise found in real data values of *r*_*h*_ greater than 0.8 have more or less the same effect on the system.

The parameter ϒ models the response to perturbations *u*. The parameter ϒ will vary in size depending on the number of input medications and vary in value depending on which variables in *x* the medications affect. With *n*_*drug*_ medications, ϒ will be a 7 × 1 + *n*_*drug*_ matrix, where the first column captures the spontaneous perturbation and the rest of the columns capture the medications. As an example, we simulate two theoretical medications for BP. ϒ in this case will have this specific structure:

$$\begin{array}{c} ϒ=[\begin{array}{ccc} r_{h} & r_{h} & r_{h}\\ r_{h}*ratio_{SD} & r_{h}*ratio_{SD} & r_{h}*ratio_{SD}\\ 0 & 0 & 0\\ 0 & 0 & 0\\ 0 & 0 & 0\\ 0 & 0 & 0\\ 0 & 0 & 0 \end{array}] \end{array}$$

Where *ratio*_*SD*_ is the ratio of diastolic to systolic effects. Because both hypothetical drugs affect only the systolic and diastolic BP, only the first two rows are non-zero. When multiplied by *u* and plugged into the full DLM *x* equation, we get the following equation for systolic BP:

$$\begin{array}{c} x_{t}^{\left(1\right)}=\left(1-r_{h}\right)*x_{t-1}^{\left(1\right)}+r_{h}*\left(x_{t-1}^{\left(3\right)}+u^{\left(1\right)}+u^{\left(2\right)}+u^{\left(3\right)}\right) \end{array}$$

In effect, the perturbations from *u* modify the homeostasis target.

The emission matrix *A* translates the true BP and HR to observed BPs and HRs. In our simplified system with only one modality of observation for each, *A* looks like the following:

$$\begin{array}{c} A=[\begin{array}{ccccccc} 1 & 0 & 0 & 0 & 0 & 0 & 0\\ 0 & 1 & 0 & 0 & 0 & 0 & 0\\ 0 & 0 & 0 & 0 & 1 & 0 & 0 \end{array}] \end{array}$$

We could add terms to *A* to capture non-zero mean errors in measurement, but for the simplicity of the model, we assume zero mean measurement errors. *A* is thus treated as known.

The covariance matrix *Q* defines the inherent variability of BP and HR as a result of perturbations to autonomic regulation, such as input and output of fluids, changes in metabolic demand, or impairment of autonomic regulation due to damage caused by an acute stroke. The rows and columns of *Q* that correspond to constants and homeostasis targets in *X* will have zero variance and covariance. We can capture the covariance between systolic and diastolic BP, as well as autonomic mechanisms that couple BP and HR variability. Other potential perturbations in the setting of stroke, such as Cushing’s response to increased intracranial pressure, are captured here in variability *Q* even if we do not explicitly model them in the perturbation model.

The covariance matrix *R* defines the measurement noise. Each method of measuring BP and HR has an inherent measurement error. For simplicity, we assume measurement noise does not vary significantly from patient to patient and thus we set *R* to be a known value.

#### Sufficiency for modeling the homeostasis perturbation model

If we set *r*_*h*_ = 1 and substitute *P*_*spon*_(*t*) for *u*^(1)^, *P*_*med*1_(*t*) for *u*^(2)^, *P*_*med*2_(*t*) for *u*^(3)^, and *SBP*_*H*_ for $x_{t-1}^{\left(3\right)}$, with unitary and known *A* and some noise *ϵ*_*Q*_ and *ϵ*_*R*_ generated from *Q* and *R*, we get Eq 3:

$$\begin{array}{cc} x_{t}^{\left(1\right)} & =\left(x_{t-1}^{\left(3\right)}+u^{\left(1\right)}+u^{\left(2\right)}+u^{\left(3\right)}\right)+\epsilon_{Q}\\ x_{t}^{\left(1\right)} & =\left(SBP_{H}+P_{spon}\left(t\right)+\sum_{meds}P_{med}\left(t\right)\right)+\epsilon_{Q}\\ SBP_{t}=y_{t}^{\left(1\right)}={\left(Ax_{t}\right)}^{\left(1\right)}+\epsilon_{R}=\left(SBP_{H}+P_{spon}\left(t\right)+\sum_{meds}P_{med}\left(t\right)\right)+\left(\epsilon_{Q}+\epsilon_{R}\right) \end{array}$$ (3)

Which is the exact homeostasis perturbation model described in Eq 1 with *ϵ*_*S*_ = *ϵ*_*Q*_ + *ϵ*_*R*_. A parallel construction can be made for DBP. Thus, we have shown that the AIM-BP model is sufficient for capturing homeostasis perturbation model parameters.

For the purposes of this paper, we are interested in estimating the following parameters of the AIM-BP model: the homeostasis targets (e.g. *x*^(3)^ or $\mu_{0}^{\left(3\right)}$ for systolic BP), the rate of decay for the stroke BP perturbation *r*_*B*_, and the drug *E*_*max*_ and *EC*_50_ parameters.

### Parameter estimation

A Metropolis-within-Gibbs sampler was written in Matlab (https://www.mathworks.com) for parameter estimation. The method by which each parameter is estimated, as well as prior used, is listed in S3 Table.

For *μ*_0_, we first estimate $\mu_{0}^{\left(1,2,5\right)}$ using a Gibbs step with a conjugate multivariate normal prior. We then estimate homeostasis targets $\mu_{0}^{\left(3,4,6\right)}$ by individual Metropolis steps, sampling from the probability:

$$\begin{array}{c} Pr\left(\mu_{0}|\Sigma_{0},\Phi ,ϒ,Q,A,R,x,x_{0},y\right)\propto Pr\left(\mu_{0}\right)Pr\left(x_{0}|\mu_{0},\Sigma_{0}\right)\prod_{t=1}^{T}Pr\left(x_{t}|x_{t-1},\Phi ,ϒ,Q,u_{t}\right) \end{array}$$

We use a normal prior for Pr(*μ*_0_). *B*_*max*_ and *ration*_*SD*_ is calculated from *μ*_0_.

For *r*_*B*_, we sample from the following probability:

$$\begin{array}{cc} & Pr(r_{B}|\mu_{0},\Sigma_{0},\Phi ,ϒ,Q,A,R,u,x,y)\\ & \propto \prod_{t=1}^{T}Pr\left(x_{t}|x_{t-1},\Phi ,ϒ,Q,u_{t}\right)Pr\left(r_{B}\right) \end{array}$$

For *E*_*max*_, and *EC*_50_, we sample from the following probability:

$$\begin{array}{cc} & Pr(E_{max},EC_{50}|\mu_{0},\Sigma_{0},\Phi ,ϒ,Q,A,R,u,x,y)\\ & \propto \prod_{t=1}^{T}Pr\left(x_{t}|x_{t-1},\Phi ,ϒ,Q,u_{t}\right)Pr\left(E_{max},EC_{50}\right) \end{array}$$

We run our MCMC sampler with a burn-in period of 2000 steps and then sample every 5 steps until we reach 2000 samples. We take the mean of the sampled values as the estimate for the parameters.

### Evaluation

The utility of the AIM-BP framework can be judged on its two major components: the AIM-BP model and the parameter estimator. Like any mathematical model of real world phenomena, the usefulness of the AIM-BP model depends on whether it is descriptive enough for its clinical application and simple enough to be reliably estimated from available data. The first part is not able to be evaluated objectively—though we believe an exponential decay model of homeostasis perturbation and an *E*_*max*_ model of drug pharmacodynamics will be sufficient to characterize how individual stroke patients’ blood pressures behave in the days after a stroke. The second part can be objectively evaluated, and can be done by evaluating the performance of the AIM-BP parameter estimator.

We evaluated the performance of our AIM-BP parameter estimator by its ability to recover three parameters of clinical importance: $\mu_{0}^{\left(3\right)}$, the systolic BP homeostasis baseline, and *E*_*max*_ and *EC*_50_ for each medication. We chose to limit the evaluation to these parameters, instead of the full set of learned parameters (S3 Table), for the purposes of clarity and simplicity.

In addition to the individual *E*_*max*_ and *EC*_50_ parameters, we also calculated an aggregate parameter we call the *E*_*max*_ ratio, which is a normalized ratio of the two separate parameters:

$$\begin{array}{c} ER=\frac{E_{max}}{EC_{5}0+mean\left(c\left(c>0\right)\right)} \end{array}$$

It has been shown that when dose-response curves do not explore the maximal dosage ranges sufficiently, individual *E*_*max*_ and *EC*_50_ estimates can be highly inaccurate but their ratio is a more stable parameter. [35] We thus introduced the *E*_*max*_ ratio parameter as a more stable, aggregate metric of drug effectiveness. A drug is more effective when its *E*_*max*_ is higher or when its *EC*_50_ is lower, so a higher *E*_*max*_ ratio corresponds to a greater measure of effectiveness. We normalized the *EC*_50_ in the denominator by adding the mean concentration of the drug during its active period (when the concentration is non-zero). This helps further stabilize the ratio.

Ideally, we would compare the performance of the AIM-BP parameter estimator against the performance of a state of the art algorithm. To our knowledge, however, we are the first to attempt to model spontaneous BP trends and medication pharmacodynamics in the critical care stroke setting simultaneously, so no such state-of-the-art algorithm exists.

While no studies have looked at our combined model, numerous studies fit pharmacodynamics parameters for the *E*_*max*_ model when studying dose-response relationships. These are usually fit using maximum likelihood estimation via a non-linear regression method such as Gauss-Newton or Levenberg-Marquardt. [13–15] We will refer to these methods as Non-Linear Least Squares (NLLS) regression methods. Instead of fitting just the *E*_*max*_ model using these methods, however, we fitted the combined perturbation model in Eqs 1 and 2. We used NLLS fitting as a state-of-the-art comparison to the AIM-BP parameter estimator.

In order to see which method performs better, estimated parameters from each method must be compared to ground truth. Unfortunately, no real world stroke dataset exists in which personalized drug pharmacodynamics parameter values are known through other means and could be used as ground truth. Furthermore, our homeostasis perturbation model is a novel characterization of spontaneous blood pressure behavior post stroke, and as such no ground truth values are available for real world data either. As such, we must use simulated data generated with ground truth parameters in order to compare the two methods. By using simulated data, we must ensure that our data generation mechanism is sufficient to model a clinically significant proportion of real data, particularly the amount of variance seen in real data. Because of this variance, one set of ground truth parameters is capable of generating a whole set of simulated time series, some of which may be more likely represented by another set of parameters. As such, our parameter estimation method must not only achieve good **accuracy**, but also must be **consistent** across multiple simulations of data using the same ground truth parameters. We will define what we mean by these terms after we discuss the data simulation methods.

#### Data simulation

As a proof of concept of the AIM-BP framework, four clinical scenarios with unique ground truth parameters were used to simulate BP data. Model parameters were learned from the simulated data using the AIM-BP parameter estimator, and the learned parameters were compared to the ground truth model parameters used to simulate the data. To generate simulated data that resembled real world data, we analyzed charted vital signs from a dataset of 497 primary intracerebral hemorrhage (ICH) patients extracted from the electronic medical record at University of Pittsburgh Medical Center (UPMC) Presbyterian Hospital (IRB PRO16110384) and 1367 ICH patients from the MIMIC III database. [29] Patient data was used to estimate, to a rough order of magnitude, the amount of variance *Q* and *R* that should be used for the simulated data.

To mitigate the effect of long term BP changes on variance calculations, variances were calculated in 6 hour long segments, after a linear fit had been subtracted. The median variances for systolic and diastolic BPs and HR as measured by BP cuff can be found in S1 Table. Since arterial BP readings are treated as the gold standard with fewer potential sources of measurement noise, we were hoping to use the difference between arterial and non-invasive variances to inform the split between inherent BP variability *Q* and measurement noise *R*. We found, however, that arterial BP variances estimated in this method were equal to or greater than non-invasive BP variances. There are several possible explanations for this: One, non-invasive BPs overestimate low BPs and underestimate high BPs compared to arterial lines. [36, 37] Two, arterial BPs are taken more frequently when a patient’s BP is more unstable. Since we are only interested in a rough order of magnitude correctness, however, we assigned *Q* and *R* arbitrarily within the order of magnitude of the variances seen.

Based on these findings, we introduced inherent BP variance *Q* in our simulation of 81*mmHg*^2^ for systolic and 49*mmHg*^2^ for diastolic BP and measurement noise variance *R* of 25*mmHg*^2^ for SBP and 16*mmHg*^2^ for DBP. HR data was not available for the UPMC dataset, so only the variance from the MIMIC dataset was used to inform our choice of *Q* and *R*. Because HR/BP interactions are not the focus of this paper, we simplified the model and did not include a covariance between those terms.

At the beginning of each ICU admission, BP data was charted roughly every 15 minutes. As such, a discrete time step of 15 minutes was chosen for the simulated data.

We crafted four different clinical scenarios that would present with similar BP time series, based on the critical care stroke BP management protocol at the neuro ICU at UPMC Presbyterian. Our goal is to demonstrate the ability of the AIM-BP framework to identify and estimate different combinations of drug effects and spontaneous BP behavior that together add to similar BP trends. The clinical scenarios all start out the same way:

A 70 year old male patient arrives at the ICU with a primary intracerebral hemorrhage. His BP at admission to the ICU is 220/110 mmHg.

The scenarios then diverge as follows:

1. Scenario 1: The patient is given an initial push of 20mg IV labetalol bolus twice to bring his systolic BP down to less than 140 mmHg. No further medication is needed to bring keep his systolic BP under 140 mmHg. Under the hood, this patient’s BP would have spontaneously trended to 130/80 mmHg, and the IV labetalol bolus was of medium effectiveness, with an *E*_*max*_ of -40 mmHg and an *EC*_50_ of 70 ng/mL.
2. Scenario 2: The patient is given the exact same push of 20mg IV labetalol bolus twice as in Scenario 1, and his BP behaves similarly. Under the hood, however, this patient’s BP would have spontaneously trended to 150/90 mmHg, but the IV labetalol bolus was particularly effective with an *E*_*max*_ of -60 mmHg and an *EC*_50_ of 40 ng/mL.
3. Scenario 3: The patient is given the exact same push of 20mg IV labetalol bolus twice as in Scenario 1, but it is not very effective. He is then started on an IV nicardipine drip, which is titrated towards a target SBP at or below 140 mmHg and only a low drip rate is needed to be effective. Under the hood, this patient’s BP would have spontaneously trended to 180/100 mmHg, the IV labetalol bolus was not very effective with an *E*_*max*_ of -20 mmHg and an *EC*_50_ of 160 ng/mL, and the IV nicardipine drip was very effective with an *E*_*max*_ of -60 mmHg and an *EC*_50_ of 40 ng/mL.
4. Scenario 4: The patient is given the exact same push of 20mg IV labetalol bolus twice as in Scenario 1, but it is not very effective. He is then started on an IV nicardipine drip, which is titrated towards a target SBP at or below 140 mmHg and a medium drip rate is needed to be effective. Under the hood, this patient’s BP would have spontaneously trended to 160/100 mmHg, the IV labetalol bolus was not very effective with an *E*_*max*_ of -20 mmHg and an *EC*_50_ of 160 ng/mL, and the IV nicardipine drip was moderately effective with an *E*_*max*_ of -40 mmHg and an *EC*_50_ of 70 ng/mL.

**Fig 4:**
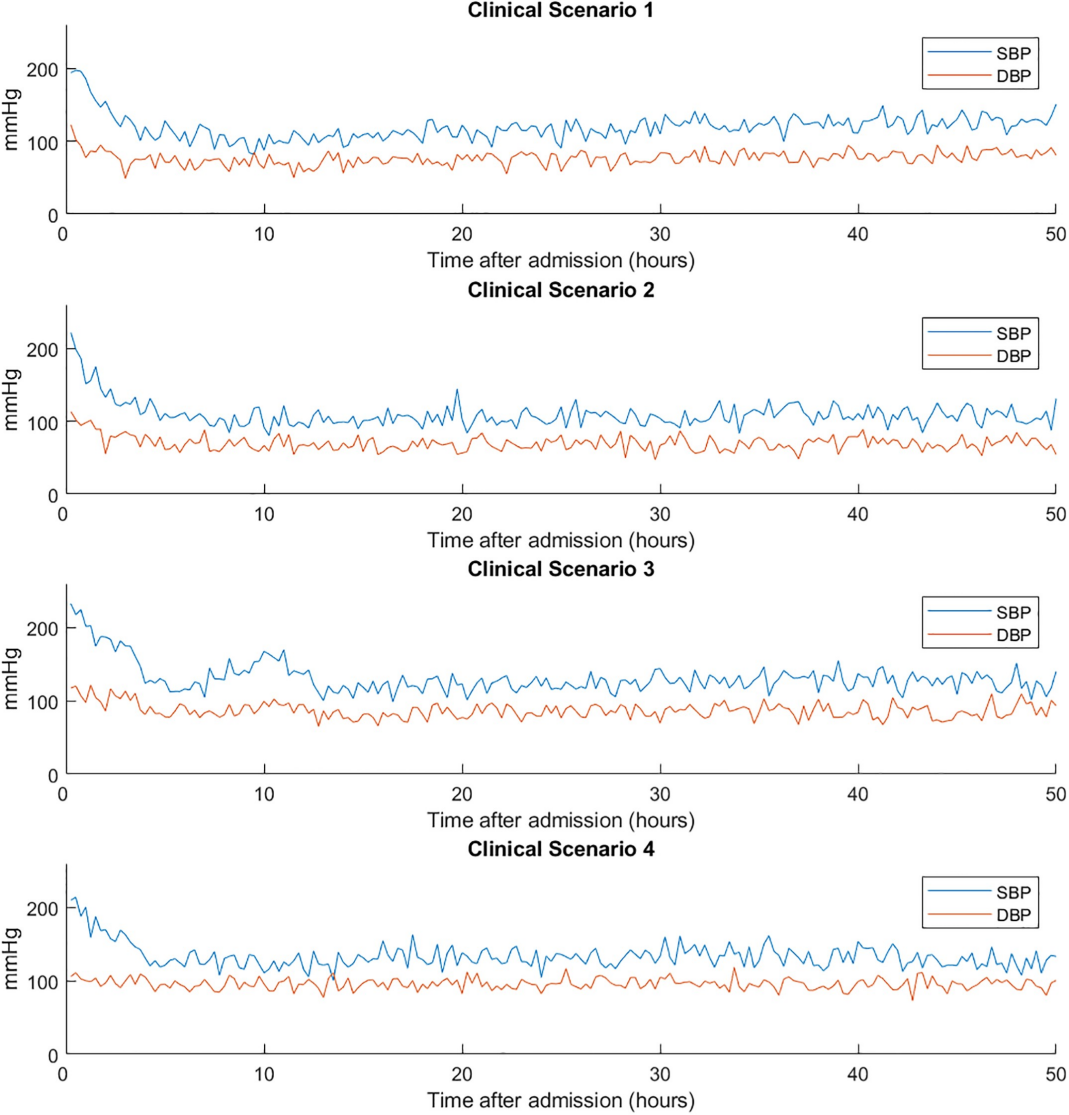
Sample simulated BP time courses for each clinical scenario. Each clinical scenario presents with a BP of around 220/110 mmHg, which is managed down to around 140 mmHg systolic using medication. Upon visual inspection, it is difficult to identify how each scenario’s patient responds differently to the medications administered to them. AIM-BP can identify the different effectiveness of medications in each scenario.

The BP chart of each clinical scenario is shown in Fig 4. These scenarios differ in three primary parameters that we will focus on: $\mu_{0}^{\left(3\right)}$, the homeostasis baseline for SBP, *E*_*max*_, the maximum effect achievable by a drug, and *EC*_50_, the drug concentration needed to achieve half of the maximum effect. The specific configurations of the relevant parameters for each clinical scenario can be found in S2 Table.

*E*_*max*_ and *EC*_50_ ranges for labetalol and nicardipine were roughly estimated from the literature. [38–45] Time to onset of peak concentration, half-life and central volumes of distribution were taken from literature or reference websites (https://www.rxlist.com, https://pubchem.ncbi.nlm.nih.gov/). Volumes of distribution, peak onset, and half-lifes were used to estimate drug plasma concentrations after doses in a rudimentary pharmacokinetics model, using the volume of distribution to calculate the peak plasma concentration, with a linear slope up to the peak for the duration of peak onset and an exponential decay after the peak concentration calculated using the half-life.

We simulated the adminstration of medication in a manner that roughly adheres to the ICH BP management guidelines at UPMC Presbyterian. In all clinical scenarios, a total of 40mg of IV labetalol in two pushes was given initially. In clinical scenarios 3 and 4, IV nicardipine was started at 5mg/hr and uptitrated 2.5mg/hr to a maximum of 15mg/hr until an SBP of 140 mmHg or lower was achieved, then decreased to 3mg/hr for maintenance with adjustments as necessary.

We chose to simulate our scenarios using 15 minute time intervals for a period of 200 time points, or a little more than 2 days. We chose to simulate for a period of 2 days as a balance between having enough time to observe patients stabilizing (factoring in drug half-lifes) and being a short enough time to be within the median length of ICU stay for a stroke patient. In the UPMC dataset, the median length of stay was 3.8 days, and 73% of all patients stayed longer than 2 days. This means that 73% of the UPMC dataset had a long enough ICU stay that generated enough data that our results on simulated data would be applicable. We leave the investigation of parameter estimation performance given varying lengths of data to future work.

#### Evaluation metrics

In order to evaluate accuracy and consistency, we compared the AIM-BP estimator to a state-of-the-art parameter fitting method using Matlab NLLS (lsqcurvefit using Levenberg-Marquardt) on Eq 1, where *SBP*_*H*_ corresponds to $\mu_{0}^{\left(3\right)}$ and other variable names are equivalent.

First, the AIM-BP data simulator was used to simulate 100 sets of data for each clinical scenario, with the corresponding parameters for the SBP homeostasis baseline and drug effects. We then ran NLLS parameter fitting method and the AIM-BP parameter estimator once on each simulated dataset to obtain a set of 100 estimated parameters for each scenario for each method.

**Fig 5:**
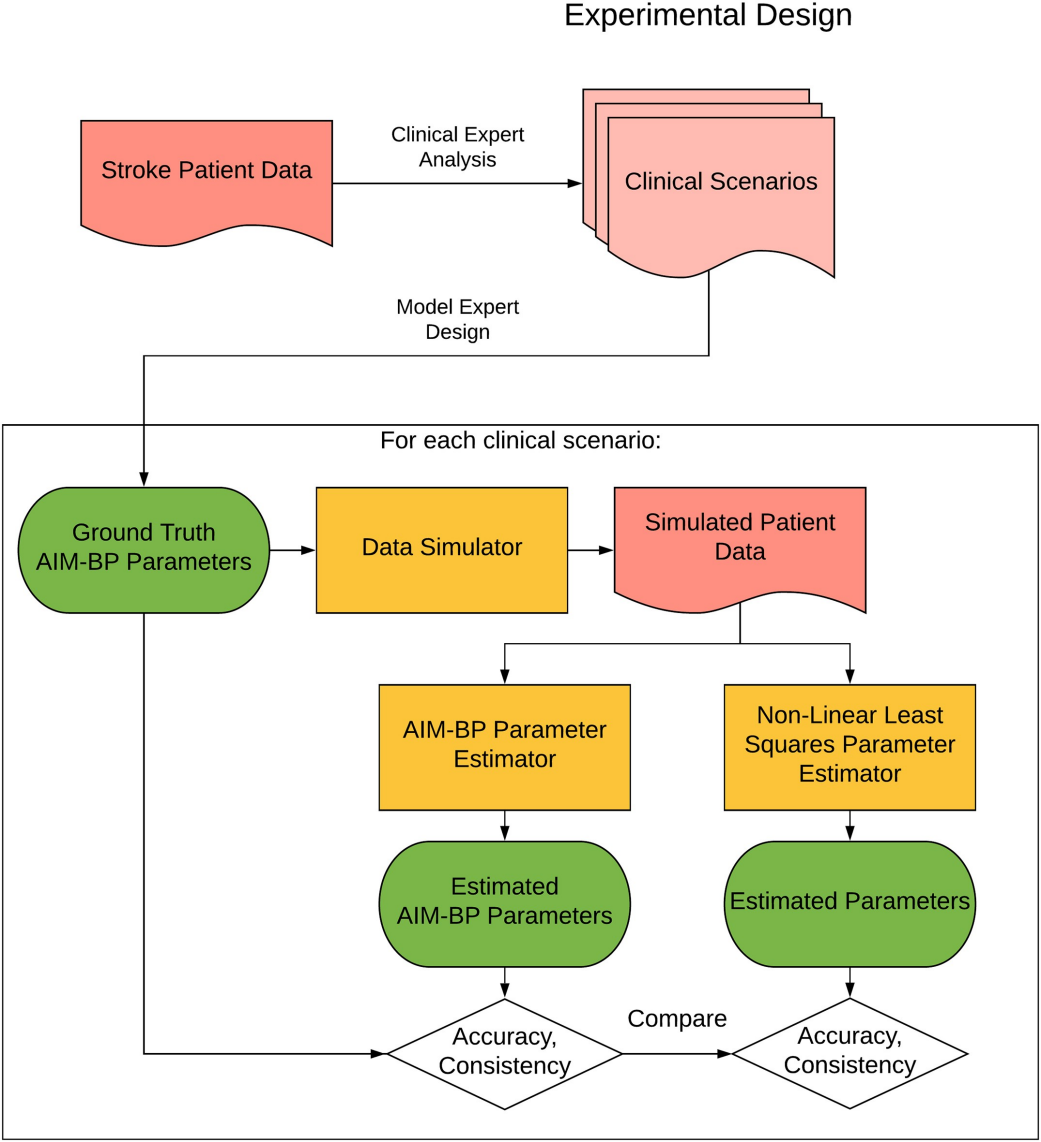
Experimental design. The accuracy and consistency of the AIM-BP parameter estimator is compared against Non-Linear Least Squares on a set of clinical scenarios.

The absolute errors of estimated parameters from each method compared to ground truth were calculated to determine if AIM-BP has on average smaller absolute error compared to NLLS, using a t-test on the paired differences of the absolute errors. **We call this metric accuracy**. Similarly, whether AIM-BP has more consistent estimates compared to NLLS was determined using an F-test on variances of estimated parameters. **We call this metric consistency**. An overview of the evaluation workflow is shown in Fig 5. In order to determine if the mechanism of data generation was responsible for performance results, we repeated the experiment for Clinical Scenarios 3 and 4, except using the homeostasis perturbation model plus noise as the data simulation mechanism. We then investigated the correlation between absolute errors from AIM-BP versus NLLS.

Finally, we identified 7 patients from the UPMC dataset who only received labetalol and/or nicardipine during their ICU stay. We used the AIM-BP parameter estimator as well as NLLS to estimate parameters for each patient. We cannot compare likelihoods for these two models in order to perform model selection, as one model estimates SBP, DBP, and HR, while the other one only estimates SBP. As such, traditional likelihood ratio tests based on maximum likelihood, AIC, or BIC are not applicable. Thus, in order to compare these models, we used both models’ estimated homeostasis perturbation models to compare residuals for systolic BP only. In order to estimate homeostasis perturbation model SBP from AIM-BP parameters, we generated data using the AIM-BP data simulated with *Q* and *R* set to 0. Calculating the homeostasis perturbation model SBP from the NLLS parameters consists of plugging in the parameters to the model. We compare residuals between the two methods to see if either is orders of magnitude worse than the other.

## Results

**Fig 6:**
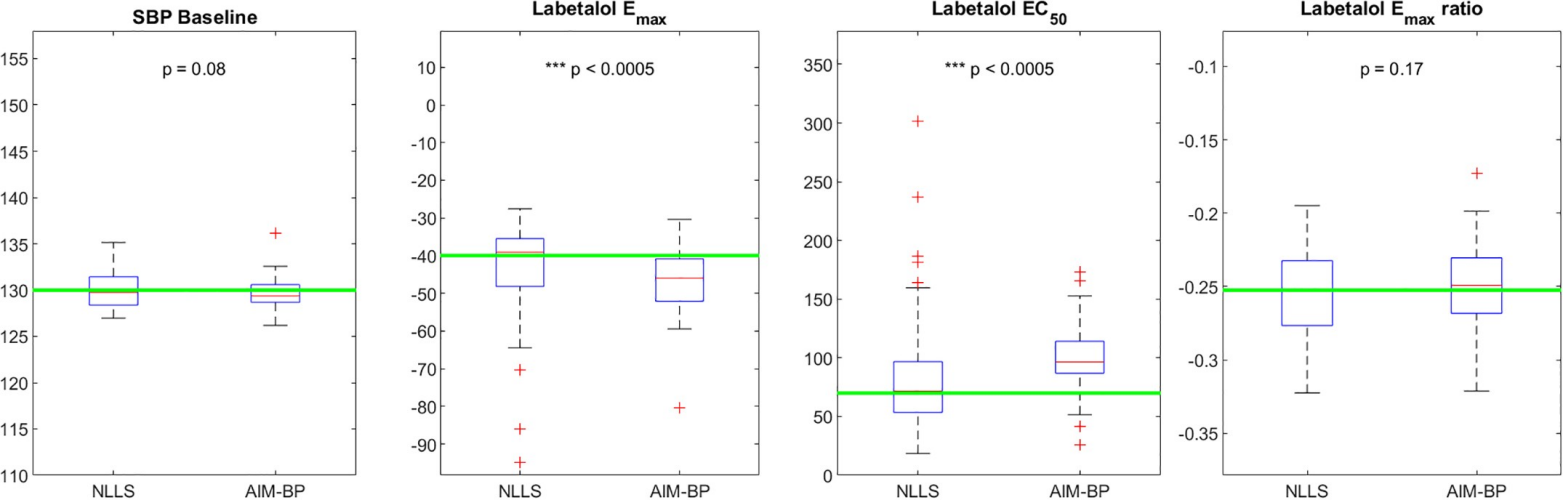
AIM-BP vs NLLS for Clinical Scenario 1. Box and whisker plots of estimated parameters using non-linear least squares (NLLS) and AIM-BP for Clinical Scenario 1. Significantly lower variances of AIM-BP estimated parameters compared to NLLS are noted with asterisks and p-values.

**Fig 7:**
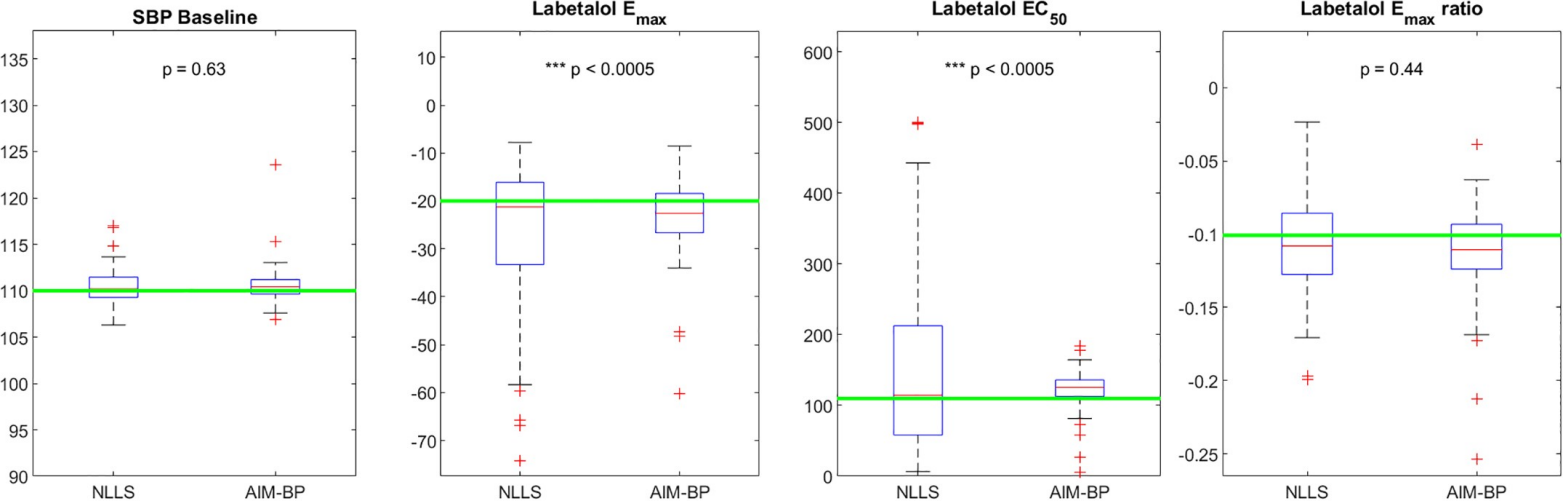
AIM-BP vs NLLS for Clinical Scenario 2. Box and whisker plots of estimated parameters using non-linear least squares (NLLS) and AIM-BP for Clinical Scenario 2. Significantly lower variances of AIM-BP estimated parameters compared to NLLS are noted with asterisks and p-values.

**Fig 8:**
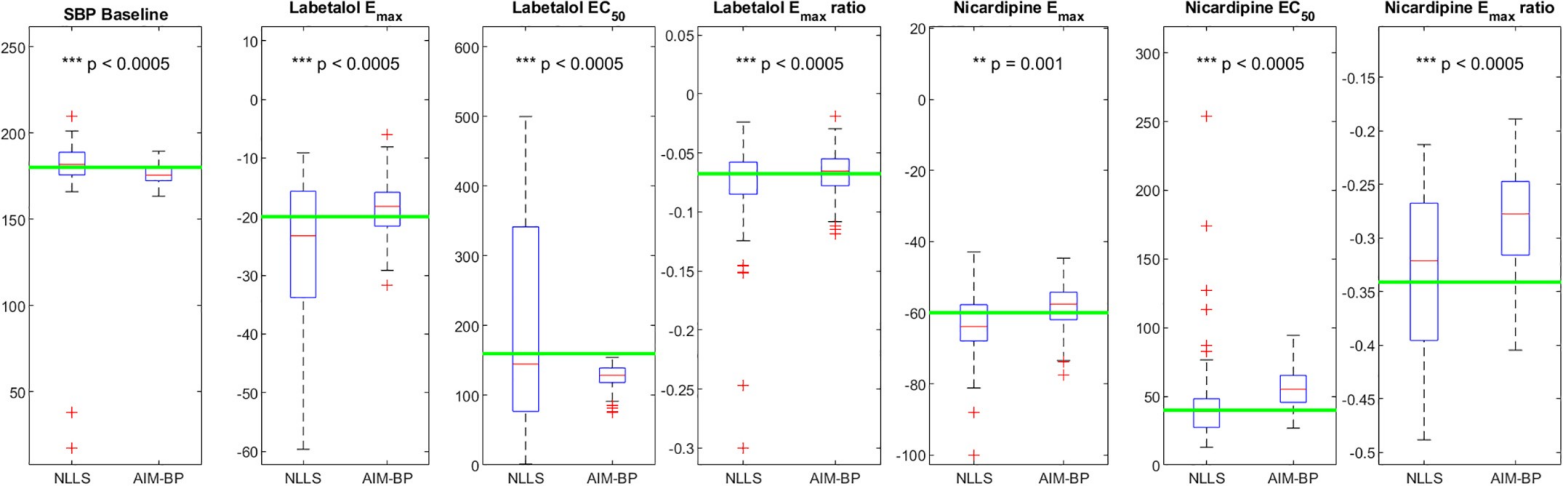
AIM-BP vs NLLS for Clinical Scenario 3. Box and whisker plots of estimated parameters using non-linear least squares (NLLS) and AIM-BP for Clinical Scenario 3. Significantly lower variances of AIM-BP estimated parameters compared to NLLS are noted with asterisks and p-values.

**Fig 9:**
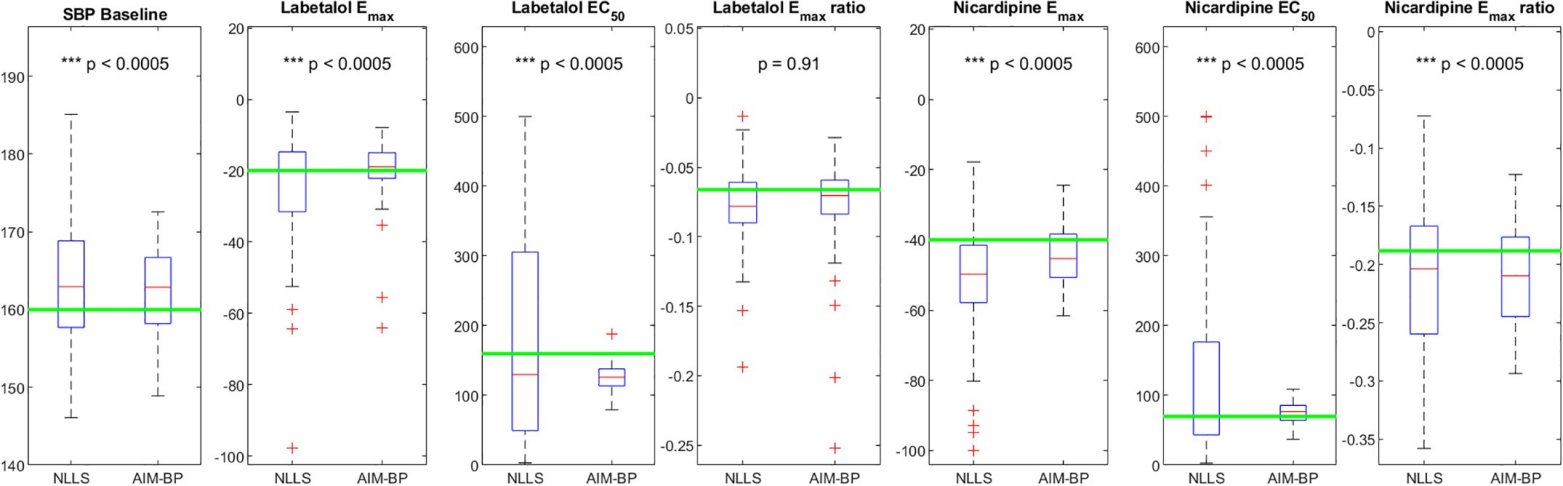
AIM-BP vs NLLS for Clinical Scenario 4. Box and whisker plots of estimated parameters using non-linear least squares (NLLS) and AIM-BP for Clinical Scenario 4. Significantly lower variances of AIM-BP estimated parameters compared to NLLS are noted with asterisks and p-values.

Accuracy results for all clinical scenarios are shown in Table 1. Consistency results for clinical scenarios are shown in Figs 6, 7, 8 and 9.

**Table 1:** Mean absolute error of estimated parameters from NLLS and AIM-BP compared to ground truth.

**Scenario 1**	**Ground Truth**	**NLLS**	**AIM-BP**
SBP homeostasis baseline ($\mu_{0}^{\left(3\right)}$)	130.0	1.5 ± 0.9	1.3 ± 1.0 *
Labetalol *E*_*max*_	−40.0	8.2 ± 8.8	7.4 ± 6.3
Labetalol *EC*_50_	70.0	30.2 ± 34.8	31.9 ± 21.2
Labetalol *E*_*max*_ ratio	−0.25	0.02 ± 0.02	0.02 ± 0.02
**Scenario 2**	**Ground Truth**	**NLLS Error**	**AIM-BP Error**
SBP homeostasis baseline ($\mu_{0}^{\left(3\right)}$)	110.0	1.3 ± 1.3	1.1 ± 1.5
Labetalol *E*_*max*_	−20.0	10.4 ± 11.1	5.3 ± 5.6 ***
Labetalol *EC*_50_	110.0	103.6 ± 110.0	22.7 ± 17.9 ***
Labetalol *E*_*max*_ ratio	−0.10	0.02 ± 0.02	0.02 ± 0.02
**Scenario 3**	**Ground Truth**	**NLLS Error**	**AIM-BP Error**
SBP homeostasis baseline ($\mu_{0}^{\left(3\right)}$)	180.0	10.1 ± 21.2	5.7 ± 4.2 *
Labetalol *E*_*max*_	−20.0	10.1 ± 8.9	3.8 ± 2.8 ***
Labetalol *EC*_50_	160.0	130.6 ± 106.4	33.3 ± 15.8 ***
Labetalol *E*_*max*_ ratio	−0.07	0.02 ± 0.03	0.01 ± 0.01 **
Nicardipine *E*_*max*_	−60.0	7.3 ± 6.2	3.8 ± 2.8 **
Nicardipine *EC*_50_	40.0	16.6 ± 27.0	17.6 ± 12.9
Nicardipine *E*_*max*_ ratio	−0.34	0.06 ± 0.03	0.06 ± 0.04
**Scenario 4**	**Ground Truth**	**NLLS Error**	**AIM-BP Error**
SBP homeostasis baseline ($\mu_{0}^{\left(3\right)}$)	160.0	7.3 ± 5.5	5.3 ± 3.4 ***
Labetalol *E*_*max*_	−20.0	10.6 ± 11.2	5.1 ± 6.1 ***
Labetalol *EC*_50_	160.0	135.7 ± 108.3	35.2 ± 17.6 ***
Labetalol *E*_*max*_ ratio	−0.07	0.02 ± 0.02	0.02 ± 0.03
Nicardipine *E*_*max*_	−40.0	15.5 ± 14.5	5.1 ± 6.1 ***
Nicardipine *EC*_50_	70.0	85.6 ± 105.0	13.8 ± 9.3 ***
Nicardipine *E*_*max*_ ratio	−0.19	0.05 ± 0.04	0.04 ± 0.03 ***

Mean and standard deviation of absolute errors from ground truth of estimated parameters from non-linear least squares (NLLS) and AIM-BP. Significantly lower AIM-BP absolute errors are noted with asterisks (*** = *p* < 0.0005, ** = *p* < 0.005, * = *p* < 0.05).

In clinical scenarios 1 and 2, both the basic non-linear least squares (NLLS) and the AIM-BP parameter estimation methods performed equally well in estimating the systolic BP baseline $\mu_{0}^{(}3)$, achieving mean absolute errors of less than 2 mmHg (Table 1). Absolute errors were significantly different for SBP baseline in Scenario 1, but by a negligible amount, and not significantly different in Scenario 2. In both scenarios, NLLS exhibited wider variability in estimating *E*_*max*_ and *EC*_50_ values, but no significant difference was seen in the *E*_*max*_ ratio parameter (Figs 6 and 7).

In the more complex two-drug clinical scenarios 3 and 4, again we see more accurate and consistent estimates for *E*_*max*_ and *EC*_50_ using AIM-BP, with the exception of the nicardipine *EC*_50_ in scenario 3 (Table 1, Fig 8) and 4 (Fig 9). Again, although we see statistically more significant consistency in SBP baseline and *E*_*max*_ ratio in some cases, the differences are not large enough to be clinically significant.

In terms of mean absolute error, AIM-BP generally consistently had smaller errors than NLLS in estimating *E*_*max*_ and *EC*_50_. For the SBP homeostasis baseline, AIM-BP was equally good (with negligibly better statistically significant performance) as NLLS in Scenarios 1 and 2, but a larger performance gap was apparent in Scenarios 3 and 4. For *E*_*max*_ ratio, both methods performed the same in all scenarios. Overall, AIM-BP performed statistically significantly better in estimating many of the parameters, but not to a degree that was clinically significant.

Because data was simulated using the AIM-BP model, one possible explanation for the increased performance of the AIM-BP parameter estimator may simply be that the data was simulated with the same model. To test if this is the case, we simulated data using just the homeostasis perturbation model plus white noise with variance *Q*^(1,2,5)^ + *R*, which is the same model upon which NLLS is fit. For clarity, we will refer to this mechanism of data simulation as the NLLS mechanism. We observed the same increase in performance for Scenarios 3 and 4 using the NLLS mechanism as when the data was simulated using an AIM-BP mechanism (Accuracy results in S4 Table and consistency results in S1 and S2 Figs).

We sought to answer the question of whether errors in estimation were correlated between the two methods. For each scenario, we plotted the absolute errors for SBP homeostasis baseline, *E*_*max*_, *EC*_50_, and *E*_*max*_ ratio of one method against the other and calculated the Pearson correlation coefficient for each parameter (S3, S4, S5 and S6 Figs for Clinical Scenarios 1-4, respectively). We observed that most variables had a moderate amount of positive correlation with *r* around 0.5. The negative or correlations close to zero were seen in *EC*_50_ estimates, where NLLS had high errors while AIM-BP did not. This degree of correlation suggests that the individual sets of simulated data themselves contribute to the error, in the sense that generally when AIM-BP had high errors, so did NLLS, and for variables that are not *EC*_50_, when NLLS had high errors, so did AIM-BP.

**Fig 10:**
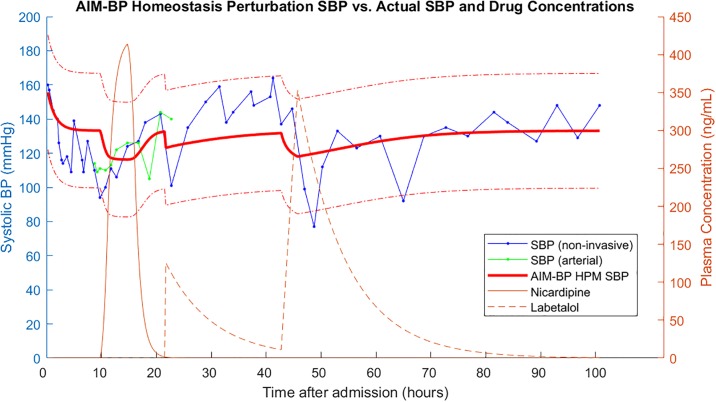
AIM-BP homeostasis perturbation model SBP compared to actual SBP and drug concentrations. Example calculated homeostasis perturbation model SBP using estimated AIM-BP parameters compared to actual SBP and drug concentrations. Dot-dashed red lines represent 2 standard deviations of *Q* from the homeostasis perturbation model SBP.

Finally, we estimated AIM-BP parameters using the AIM-BP parameter estimator and homeostasis perturbation model parameters using NLLS on 7 patients in the UPMC ICH dataset that only used labetalol or nicardipine for blood pressure management. An example AIM-BP fitted homeostasis perturbation model systolic BP is shown in Fig 10. The residuals of the calculated AIM-BP homeostasis perturbation model SBP were compared against the residuals of the NLLS homeostasis perturbation model SBP in Table 2. NLLS is slightly better in 4 patients and AIM-BP is slightly better in 3 patients, but neither is orders of magnitude worse.

**Table 2:** Residuals of AIM-BP vs NLLS homeostasis perturbation models on 7 UPMC ICH patients.

Patient	NLLS	AIM-BP
1	28909.9	27006.0
2	10726.1	10371.3
3	39778.4	45074.0
4	33563.5	33916.3
5	18442.2	18784.3
6	23045.7	22859.4
7	34760.4	36461.1

Residuals are calculated by taking the sum of squares of homeostasis perturbation model SBP minus actual measured SBP. If both a non-invasive BP and an arterial line were measured at a given time point, both residuals were added.

## Discussion

We evaluated the ability of our Acute Intervention Model of Blood Pressure (AIM-BP) to identify drug effectiveness and parameters that govern spontaneous BP behavior post hemorrhagic stroke. We simulated 4 clinical scenarios with ground truth parameters. We compared AIM-BP performance on these 4 scenarios to a state-of-the-art maximum likelihood estimation method using non-linear least squares curve fitting. Compared to ground truth parameter values, AIM-BP produced equal or smaller mean absolute errors than NLLS in the more complex scenarios 3 and 4, and performed equally well in the simple scenarios 1 and 2. Specifically, AIM-BP had more accurate and consistent estimates for *E*_*max*_ and *EC*_50_ parameters. This increased accuracy was likely due to the use of prior distributions on *E*_*max*_ and *EC*_50_, which limits the number of combinations that can produce similar drug effects. Notably, this increase in accuracy largely disappears when we look at the transformed variable *E*_*max*_ ratio. In addition, AIM-BP was more consistent in estimating parameter values, especially in the more complex scenarios that involve multiple drugs. Finally, in estimating homeostasis perturbation models on 7 real world intracerebral hemorrhage patients, NLLS and AIM-BP achieved similar residuals, despite AIM-BP also fitting to DBP data. The number of patients we fitted models to was limited by the drugs we researched pharmacokinetics data and pharmacodynamics priors for. In future work, we plan to leverage data from DrugBank (https://www.drugbank.ca) to auto-populate pharmacokinetics information and pharmacodynamics priors for more drugs.

As with the choice of any model, there is a trade-off in AIM-BP between model complexity and the full scope of what the model can capture. The assumptions that our spontaneous perturbation model makes are as follows: One, that there is some initial perturbation from homeostasis seen at admission (whether that perturbation be positive, negative, or zero), and two that a patient’s BP trends towards a constant homeostasis level. What this model allows for is a variety of trajectories, including both increases and decreases in BP, and a fast return to homeostasis vs a more gradual return that more closely resembles a constant decrease. What this model does not explicitly capture are things like cyclic effects (e.g. sleep-wake cycle), or time periods where a patient is acutely decompensating (e.g. if a patient develops symptoms of shock). We can expand the flexibility of the spontaneous perturbation model by adding components that reflect cyclic effects or replacing it with a piece-wise model with separate parameters for individual time periods. These approaches increase model complexity by increasing the number of parameters, and thus an increased amount of data will be required to accurately learn model parameters.

A major limiting factor in our model design as it stands is the translation of medication dosages into estimates of active concentrations at each time point. While this can theoretically be calculated from information on the pharmacokinetics of the drug available from the manufacturer, in reality differences in individual metabolic and clearance rates mean the active drug concentrations at a given time point will differ on a patient to patient basis even if the dosages given were the same. In theory, these variations are yet another parameter that may be learned using our MCMC-based parameter estimator framework. However, issues with the accuracy of timestamps for medication administration in the ICU may limit the usefulness of a more detailed modeling system. These issues can be circumvented in a research study setting if drug plasma concentration is measured regularly.

Real world medical chart data often contains irregularly recorded or missing data. In our neuro ICU dataset, measurements of arterial BP or non-invasive BP were recorded at irregular intervals. Charting frequency was generally higher at the beginning of an admission, dropping down to about once an hour once the patient is stable, then dropping down to once every several hours later on in the admission. Occasionally, gaps can arise due to transfer of care from the ICU, such as when a patient needs to undergo a surgery or other procedure. This missing data will need to be accounted for when feeding ICU chart data into the AIM-BP parameter estimator. Because data will need to be converted into discrete time steps, a combination of interpolation and the inclusion of missing data will be needed. Fortunately, dynamic linear models inherently handle missing data well, as the methods calculate the best estimate for hidden variables whether or not an observation was made at a given time point. In addition, it handles measurements of BP from multiple modalities (e.g. both arterial line and pressure cuff). Although we have not simulated multi-modal data in this paper, the AIM-BP system can be easily adapted to handle it by changing the *A* emission matrix. By using a DLM, we can combine multiple measurement modalities for a more accurate estimate of a patient’s true underlying vital signs.

We have limited our current evaluation of the AIM-BP model to only parameters that are directly comparable to those using a NLLS method. In future work, we plan to expand evaluation to other parts of the AIM-BP model that have no direct correlate in the homeostasis perturbation model. The parameters we investigated in this paper are not the only ones that may have clinical significance. One possible clinical parameter of interest is the inherent BP variability of a patient. Short-term BP variability has been associated with adverse cardiovascular events such as stroke. [46, 47] BP variability has been used as a feature in the prediction of sepsis. [48] Instead of looking at BP variability as an aggregate mean and standard deviation over time, the AIM-BP framework separates out external perturbatory effects and measurement noise and captures BP variability in the covariance matrix *Q*. In addition, by learning the parameter *r*_*h*_ in Φ that governs rate of return to homeostasis, AIM-BP can characterize the responsiveness of a patient’s innate homeostatic drive, which may be an additional measure of variability and a novel “vital sign” in ICU patients. By using the AIM-BP framework to estimate these parameters, we can leverage multi-modal measurements of BP to increase accuracy of variability measures, as well as describe variability in a multivariate sense. AIM-BP would provide a more robust method of investigating differences in BP variablity compared to simple variance calculations.

## Conclusion

We constructed and tested the Acute Intervention Model of Blood Pressure (AIM-BP), a personalized dynamic linear model-based framework for studying the effects of BP management in the critical care setting. We built the AIM-BP framework and tested its ability to estimate clinically important parameters using four simulated clinical scenarios. We showed that the AIM-BP framework is sufficient for capturing parameters of a BP homeostasis perturbation model. We found that the AIM-BP parameter estimator is as accurate and consistent in estimating the homeostasis perturbation model parameters as a state-of-the-art maximum likelihood estimation method, and more accurate and consistent in estimating specific *E*_*max*_ and *EC*50 parameters. By doing so, we have demonstrated the feasibility of learning quantitative pharmacodynamics and disease perturbation parameters from clinical data. Future work will evaluate the performance of the AIM-BP framework with missing and multi-modal data.

## Supporting information

S1 Table Median variances of arterial SBP, DBP, and HR. (PDF)

S2 Table Specific parameter configurations for each clinical scenario. (PDF)

S3 Table AIM-BP parameters and the method and priors used in their estimation. (PDF)

S4 Table Mean absolute error of estimated parameters from NLLS and AIM-BP compared to ground truth using NLLS simulation mechanism. (PDF)

S1 Fig Clinical Scenario 3 with NLLS simulation mechanism. Box and whisker plots of estimated parameters using non-linear least squares (NLLS) and AIM-BP for Clinical Scenario 3. Significantly lower variances of AIM-BP estimated parameters compared to NLLS are noted with asterisks and p-values.
(TIFF)

S2 Fig Clinical Scenario 4 with NLLS simulation mechanism. Box and whisker plots of estimated parameters using non-linear least squares (NLLS) and AIM-BP for Clinical Scenario 4. Significantly lower variances of AIM-BP estimated parameters compared to NLLS are noted with asterisks and p-values.
(TIFF)

S3 Fig Absolute error correlation for Clinical Scenario 1. Absolute errors of SBP homeostasis baseline, *E*_*max*_, *EC*_50_, and *E*_*max*_ ratio from AIM-BP estimates were plotted against absolute errors from NLLS estimates for Scenario 1. A Pearson correlation coefficient was calculated for each.
(TIFF)

S4 Fig Absolute error correlation for Clinical Scenario 2. Absolute errors of SBP homeostasis baseline, *E*_*max*_, *EC*_50_, and *E*_*max*_ ratio from AIM-BP estimates were plotted against absolute errors from NLLS estimates for Scenario 2. A Pearson correlation coefficient was calculated for each.
(TIFF)

S5 Fig Absolute error correlation for Clinical Scenario 3. Absolute errors of SBP homeostasis baseline, *E*_*max*_, *EC*_50_, and *E*_*max*_ ratio from AIM-BP estimates were plotted against absolute errors from NLLS estimates for Scenario 3. A Pearson correlation coefficient was calculated for each.
(TIFF)

S6 Fig Absolute error correlation for Clinical Scenario 4. Absolute errors of SBP homeostasis baseline, *E*_*max*_, *EC*_50_, and *E*_*max*_ ratio from AIM-BP estimates were plotted against absolute errors from NLLS estimates for Scenario 4. A Pearson correlation coefficient was calculated for each.
(TIFF)

## References

[1] Bowry R, Navalkele DD, Gonzales NR. Blood pressure management in stroke: Five new things. Neurology Clinical practice. 2014;4(5):419–426. 10.1212/CPJ.0000000000000085 25317377 PMC4196458

[2] Alqadri SL, Sreenivasan V, Qureshi AI. Acute Hypertensive Response Management in Patients with Acute Stroke. Current Cardiology Reports. 2013;15(12):426 10.1007/s11886-013-0426-7 24142579

[3] Butcher K, Selim M. Acute Blood Pressure Management in Intracerebral Hemorrhage: Equipoise Resists an Attack. Stroke. 2016;47(12):3065–3066. 10.1161/STROKEAHA.116.015060 27895301 PMC5134899

[4] Jauch EC, Saver JL, Adams HP, Bruno A, Connors JJB, Demaerschalk BM, et al Guidelines for the Early Management of Patients With Acute Ischemic Stroke. Stroke. 2013;44(3). 10.1161/STR.0b013e318284056a 23370205

[5] ENOS Trial Investigators. Efficacy of nitric oxide, with or without continuing antihypertensive treatment, for management of high blood pressure in acute stroke (ENOS): a partial-factorial randomised controlled trial. Lancet (London, England). 2015;385(9968):617–628. 10.1016/S0140-6736(14)61121-1 PMC4343308 25465108

[6] Castillo J, Leira R, García MM, Serena J, Blanco M, Dávalos A. Blood Pressure Decrease During the Acute Phase of Ischemic Stroke Is Associated With Brain Injury and Poor Stroke Outcome. Stroke. 2004;35(2):520–526. 10.1161/01.STR.0000109769.22917.B0 14726553

[7] McManus M, Liebeskind DS. Blood Pressure in Acute Ischemic Stroke. Journal of clinical neurology (Seoul, Korea). 2016;12(2):137–46. 10.3988/jcn.2016.12.2.137 PMC4828558 26833984

[8] Powers WJ, Rabinstein AA, Ackerson T, Adeoye OM, Bambakidis NC, Becker K, et al 2018 Guidelines for the Early Management of Patients With Acute Ischemic Stroke: A Guideline for Healthcare Professionals From the American Heart Association/American Stroke Association. Stroke. 2018;49(3):e46–e110. 10.1161/STR.0000000000000158 29367334

[9] Adams HP, del Zoppo G, Alberts MJ, Bhatt DL, Brass L, Furlan A, et al Guidelines for the Early Management of Adults With Ischemic Stroke. Circulation. 2007;115(20). 10.1161/CIRCULATIONAHA.107.181486 17515473

[10] Strbian D, Saposnik G. Review of the ENCHANTED Trial (Enhanced Control of Hypertension and Thrombolysis Stroke Study): How Low Can We Go With Intravenous Tissue-Type Plasminogen Activator Dose and Blood Pressure Level? Stroke. 2016;47(12):3063–3064. 10.1161/STROKEAHA.116.015594 27789665

[11] Anderson CS, Robinson T, Lindley RI, Arima H, Lavados PM, Lee TH, et al Low-Dose versus Standard-Dose Intravenous Alteplase in Acute Ischemic Stroke. New England Journal of Medicine. 2016;374(24):2313–2323. 10.1056/NEJMoa1515510 27161018

[12] Hemphill JC, Greenberg SM, Anderson CS, Becker K, Bendok BR, Cushman M, et al Guidelines for the Management of Spontaneous Intracerebral Hemorrhage: A Guideline for Healthcare Professionals From the American Heart Association/American Stroke Association. Stroke. 2015;46(7):2032–60. 10.1161/STR.0000000000000069 26022637

[13] Wu J, Banerjee A, Jin B, Menon SM, Martin SW, Heatherington AC. Clinical dose-response for a broad set of biological products: A model-based meta-analysis. Statistical Methods in Medical Research. 2017; p. 096228021668452. 10.1177/0962280216684528 28067121

[14] Macdougall J. Analysis of Dose-Response Studies-Emax Model In: Dose Finding in Drug Development. New York, NY: Springer New York; 2006 p. 127–145. Available from: http://link.springer.com/10.1007/0-387-33706-7{_}9.

[15] Marquardt DW. An Algorithm for Least-Squares Estimation of Nonlinear Parameters. Journal of the Society for Industrial and Applied Mathematics. 1963;11(2):431–441. 10.1137/0111030

[16] Wallace JD, Levy LL. Blood pressure after stroke. JAMA. 1981;246(19):2177–80. 10.1001/jama.1981.03320190035023 7289008

[17] Britton M, Carlsson A, de Faire U. Blood pressure course in patients with acute stroke and matched controls. Stroke. 1986;17(5):861–4. 10.1161/01.str.17.5.861 3764955

[18] Willmot M, Leonardi-Bee J, Bath PMW. High blood pressure in acute stroke and subsequent outcome: a systematic review. Hypertension (Dallas, Tex: 1979). 2004;43(1):18–24. 10.1161/01.HYP.0000105052.65787.35 14662649

[19] Appleton JP, Sprigg N, Bath PM. Blood pressure management in acute stroke. BMJ. 2016;1(2):72–82. 10.1136/svn-2016-000020 PMC5435190 28959467

[20] Liu Z, Hauskrecht M. A Regularized Linear Dynamical System Framework for Multivariate Time Series Analysis. Proceedings of the AAAI Conference on Artificial Intelligence AAAI Conference on Artificial Intelligence. 2015;2015:1798–1804. 25905027 PMC4402162

[21] Liu Z, Hauskrecht M. Learning Adaptive Forecasting Models from Irregularly Sampled Multivariate Clinical Data. Proceedings of the AAAI Conference on Artificial Intelligence AAAI Conference on Artificial Intelligence. 2016;2016:1273–1279. 27525189 PMC4980099

[22] Lau EHY, Cheng CKY, Ip DKM, Cowling BJ. Situational Awareness of Influenza Activity Based on Multiple Streams of Surveillance Data Using Multivariate Dynamic Linear Model. PLoS ONE. 2012;7(5):e38346 10.1371/journal.pone.0038346 22675456 PMC3364986

[23] Petris G, Petrone S, Campagnoli P. Dynamic linear models BT—Dynamic Linear Models with R. New York, NY: Springer New York; 2009 p. 31–84. Available from: 10.1007/b135794{_}2.

[24] Raphan T, Cohen B, Xiang Y, Yakushin SB. A Model of Blood Pressure, Heart Rate, and Vaso-Vagal Responses Produced by Vestibulo-Sympathetic Activation. Frontiers in neuroscience. 2016;10:96 10.3389/fnins.2016.00096 27065779 PMC4814511

[25] Li Q, Mark RG, Clifford GD. Artificial arterial blood pressure artifact models and an evaluation of a robust blood pressure and heart rate estimator. BioMedical Engineering OnLine. 2009;8(1):13 10.1186/1475-925X-8-13 19586547 PMC2728101

[26] Lehman LwH, Nemati S, Adams RP, Moody G, Malhotra A, Mark RG. Tracking progression of patient state of health in critical care using inferred shared dynamics in physiological time series. Conference proceedings: Annual International Conference of the IEEE Engineering in Medicine and Biology Society IEEE Engineering in Medicine and Biology Society Annual Conference. 2013;2013:7072–5. 10.1109/EMBC.2013.6611187 PMC4030703 24111374

[27] Lehman LwH, Adams RP, Mayaud L, Moody GB, Malhotra A, Mark RG, et al A Physiological Time Series Dynamics-Based Approach to Patient Monitoring and Outcome Prediction. IEEE Journal of Biomedical and Health Informatics. 2015;19(3):1068–1076. 10.1109/JBHI.2014.2330827 25014976 PMC4346516

[28] Lehman LwH, Mark RG, Nemati S. A Model-Based Machine Learning Approach to Probing Autonomic Regulation From Nonstationary Vital-Sign Time Series. IEEE Journal of Biomedical and Health Informatics. 2018;22(1):56–66. 10.1109/JBHI.2016.2636808 27959829 PMC5896770

[29] Johnson AEW, Pollard TJ, Shen L, Lehman LwH, Feng M, Ghassemi M, et al MIMIC-III, a freely accessible critical care database. Scientific Data. 2016;3:160035 10.1038/sdata.2016.35 27219127 PMC4878278

[30] Yapps B, Shin S, Bighamian R, Thorsen J, Arsenault C, Quraishi SA, et al Hypotension in ICU Patients Receiving Vasopressor Therapy. Scientific Reports. 2017;7(1):8551 10.1038/s41598-017-08137-0 28819101 PMC5561088

[31] Carlin BP, Polson NG, Stoffer DS. A Monte Carlo Approach to Nonnormal and Nonlinear State-Space Modeling. Journal of the American Statistical Association. 1992;87(418):493 10.1080/01621459.1992.10475231

[32] Carter CK, Kohn R. On Gibbs sampling for state space models. Biometrika. 1994;81(3):541–553. 10.1093/biomet/81.3.541

[33] Fearnhead P. In: Brooks S, Gelman A, Jones G, Meng XL, editors. MCMC for state-space models Chapman & Hall/CRC Handbooks of Modern Statistical Methods. Chapman and Hall; 2011 p. 513–529.

[34] Mahdi A, Sturdy J, Ottesen JT, Olufsen MS. Modeling the Afferent Dynamics of the Baroreflex Control System. PLoS Computational Biology. 2013;9(12):e1003384 10.1371/journal.pcbi.1003384 24348231 PMC3861044

[35] Schoemaker RC, van Gerven JM, Cohen AF. Estimating potency for the Emax-model without attaining maximal effects. Journal of pharmacokinetics and biopharmaceutics. 1998;26(5):581–93. 10.1023/A:1023277201179 10205772

[36] Ribezzo S, Spina E, Di Bartolomeo S, Sanson G. Noninvasive techniques for blood pressure measurement are not a reliable alternative to direct measurement: a randomized crossover trial in ICU. TheScientificWorldJournal. 2014;2014:353628 10.1155/2014/353628 24616624 PMC3926274

[37] Lehman LwH, Saeed M, Talmor D, Mark R, Malhotra A. Methods of Blood Pressure Measurement in the ICU*. Critical Care Medicine. 2013;41(1):34–40. 10.1097/CCM.0b013e318265ea46 23269127 PMC3724452

[38] Lalonde RL, O’Rear TL, Wainer IW, Drda KD, Herring VL, Bottorff MB. Labetalol pharmacokinetics and pharmacodynamics: Evidence of stereoselective disposition. Clinical Pharmacology and Therapeutics. 1990;48(5):509–519. 10.1038/clpt.1990.187 2225711

[39] Saotome T, Minoura S, Terashi K, Sato T, Echizen H, Ishizaki T. Labetalol in Hypertension During the Third Trimester of Pregnancy: Its Antihypertensive Effect and Pharmacokinetic-Dynamic Analysis. The Journal of Clinical Pharmacology. 1993;33(10):979–988. 10.1002/j.1552-4604.1993.tb01933.x 8227470

[40] Chauvin M, Deriaz H, Viars P. Continuous i.v. infusion of labetalol for postoperative hypertension. Haemodynamic effects and plasma kinetics. British journal of anaesthesia. 1987;59(10):1250–6. 10.1093/bja/59.10.1250 3676053

[41] Wilson DJ, Wallin JD, Vlachakis ND, Freis ED, Vidt DG, Michelson EL, et al Intravenous labetalol in the treatment of severe hypertension and hypertensive emergencies. The American journal of medicine. 1983;75(4A):95–102. 10.1016/0002-9343(83)90141-9 6139020

[42] Serlin MJ, Orme MC, Maciver M, Green GJ, Macnee CM, Breckenridge AM. Rate of onset of hypotensive effect of oral labetalol. British journal of clinical pharmacology. 1979;7(2):165–8. 10.1111/j.1365-2125.1979.tb00916.x 760748 PMC1429443

[43] Richards DA, Maconochie JG, Bland RE, Hopkins R, Woodings EP, Martin LE. Relationship between plasma concentrations and pharmacological effects of labetalol. European Journal of Clinical Pharmacology. 1977;11(2):85–90. 10.1007/BF00562897 14010

[44] Curran MP, Robinson DM, Keating GM. Intravenous nicardipine: its use in the short-term treatment of hypertension and various other indications. Drugs. 2006;66(13):1755–82. 10.2165/00003495-200666130-00010 16978041

[45] Graham DJ, Dow RJ, Hall DJ, Alexander OF, Mroszczak EJ, Freedman D. The metabolism and pharmacokinetics of nicardipine hydrochloride in man. British journal of clinical pharmacology. 1985;20 Suppl 1(Suppl 1):23S–28S. 10.1111/j.1365-2125.1985.tb05141.x 4027149 PMC1400776

[46] Mancia G. Short- and Long-Term Blood Pressure Variability: Present and Future. Hypertension. 2012;60(2):512–517. 10.1161/HYPERTENSIONAHA.112.194340 22733459

[47] Stevens SL, Wood S, Koshiaris C, Law K, Glasziou P, Stevens RJ, et al Blood pressure variability and cardiovascular disease: systematic review and meta-analysis. BMJ (Clinical research ed). 2016;354:i4098. 10.1136/bmj.i4098 PMC4979357 27511067

[48] de Castilho FM, Ribeiro ALP, da Silva JLP, Nobre V, de Sousa MR. Heart rate variability as predictor of mortality in sepsis: A prospective cohort study. PloS one. 2017;12(6):e0180060 10.1371/journal.pone.0180060 28654692 PMC5487061

