# Supplementary material for: Towards precision critical care management of blood pressure in hemorrhagic stroke patients using dynamic linear models
Source: PLoS One. 2019 Aug 5;14(8):e0220283. doi: 10.1371/journal.pone.0220283 (PMC6681940; doi:10.1371/journal.pone.0220283)
Supplement: S1 Table — (PDF) [file pone.0220283.s001.pdf]

S1 Table: Median variances of arterial SBP, DBP, and HR.

Variable	UPMC Median Variance	MIMIC Median Variance
Systolic BP (Non-Invasive)	113.87	67.87
Diastolic BP (Non-Invasive)	65.29	38.10
Systolic BP (Arterial)	113.96	89.98
Diastolic BP (Arterial)	22.81	21.98
HR	N/A	15.63

Median variances were calculated over all ischemic stroke patients in 6 hour segments.
