# Supplementary material for: Towards precision critical care management of blood pressure in hemorrhagic stroke patients using dynamic linear models
Source: PLoS One. 2019 Aug 5;14(8):e0220283. doi: 10.1371/journal.pone.0220283 (PMC6681940; doi:10.1371/journal.pone.0220283)
Supplement: S2 Table — (PDF) [file pone.0220283.s002.pdf]

S2 Table: Specific parameter configurations for each clinical scenario.

Parameter	Scenario 1	Scenario 2	Scenario 3	Scenario 4
Initial SBP ($\mu_0^{(1)}$)	220 mmHg			
SBP Homeostasis Base ($\mu_0^{(3)}$)	130 mmHg	110 mmHg	180 mmHg	160 mmHg
Labetalol E_{max}	-40 mmHg	-20 mmHg	-20 mmHg	-20 mmHg
Labetalol EC_{50}	70 ng/mL	110 ng/mL	160 ng/mL	160 ng/mL
Nicardipine E_{max}	N/A	N/A	-60 mmHg	-40 mmHg
Nicardipine EC_{50}	N/A	N/A	40 ng/mL	70 ng/mL
Initial DBP ($\mu_0^{(2)}$)	110 mmHg			
DBP Homeostasis Base ($\mu_0^{(4)}$)	80 mmHg	70 mmHg	100 mmHg	100 mmHg
Initial HR ($\mu_0^{(5)}$)	70 bpm			
HR Homeostasis Base ($\mu_0^{(6)}$)	70 bpm			
Spontaneous perturbation decay rate (r_B)	0.1			
BP homeostasis rate (r_h)	0.9			
