# Supplementary material for: Towards precision critical care management of blood pressure in hemorrhagic stroke patients using dynamic linear models
Source: PLoS One. 2019 Aug 5;14(8):e0220283. doi: 10.1371/journal.pone.0220283 (PMC6681940; doi:10.1371/journal.pone.0220283)
Supplement: S3 Table — (PDF) [file pone.0220283.s003.pdf]

S3 Table: AIM-BP parameters and the method and priors used in their estimation.

Parameter	Estimation Method	Prior
X, X_0	Forward Filtering Backward Sampling algorithm [1]	None
$\mu_0^{(1,2,5)}$	Gibbs step	Conjugate $MVN(\begin{bmatrix} 220 \\ 110 \\ 70 \end{bmatrix}, \begin{bmatrix} 0.1 & 0 & 0 \\ 0 & 0.1 & 0 \\ 0 & 0 & 0.1 \end{bmatrix})$
$\mu_0^{(3,4,6)}$	Metropolis step	$N(140, 1600)$<br>$N(90, 900)$<br>$N(70, 100)$
$\mu_0^{(7)}$	Constant and known	None
Σ_0	Constant and known	None
r_B	Metropolis step	$Unif(0.005, 0.2)$
B_{max}	Derived from μ_0	None
E_{max}, EC_{50}	Metropolis step	Labetalol: $MVN(\begin{bmatrix} -30 \\ 110 \end{bmatrix}, \begin{bmatrix} 900 & 0 \\ 0 & 2500 \end{bmatrix})$<br>Nicardipine: $MVN(\begin{bmatrix} -30 \\ 70 \end{bmatrix}, \begin{bmatrix} 900 & 0 \\ 0 & 900 \end{bmatrix})$
Σ_0	Constant and known	None
Φ	Constant and known	None
Υ	Derived from B_{max} and μ_0	None
Q	Constant and known	None
A	Constant and known	None
R	Constant and known	None

References

1. Frühwirth-Schnatter S. Data Augmentation and Dynamic Linear Models. Journal of Time Series Analysis. 1994;15(2):183–202. doi:10.1111/j.1467-9892.1994.tb00184.x.
