# Supplementary material for: Towards precision critical care management of blood pressure in hemorrhagic stroke patients using dynamic linear models
Source: PLoS One. 2019 Aug 5;14(8):e0220283. doi: 10.1371/journal.pone.0220283 (PMC6681940; doi:10.1371/journal.pone.0220283)
Supplement: S4 Table — (PDF) [file pone.0220283.s004.pdf]

S4 Table: Mean absolute error of estimated parameters from NLLS and AIM-BP compared to ground truth using NLLS simulation mechanism.

**Scenario 3**	**Ground Truth**	**NLLS Error**	**AIM-BP Error**
SBP homeostasis baseline ($\mu_0^{(3)}$)	180.0	12.1 ± 27.4	5.1 ± 3.6 *
Labetalol E_{max}	-20.0	10.1 ± 9.0	4.0 ± 3.2 ***
Labetalol EC_{50}	160.0	139.4 ± 106.5	32.3 ± 16.0 ***
Labetalol E_{max} ratio	-0.07	0.02 ± 0.02	0.02 ± 0.01 ***
Nicardipine E_{max}	-60.0	7.9 ± 6.4	4.0 ± 3.2 ***
Nicardipine EC_{50}	40.0	17.3 ± 20.1	18.0 ± 13.9
Nicardipine E_{max} ratio	-0.34	0.06 ± 0.04	0.06 ± 0.04
**Scenario 4**	**Ground Truth**	**NLLS Error**	**AIM-BP Error**
SBP homeostasis baseline ($\mu_0^{(3)}$)	160.0	7.6 ± 16.1	4.1 ± 3.4 *
Labetalol E_{max}	-20.0	11.2 ± 12.3	6.3 ± 6.7 ***
Labetalol EC_{50}	160.0	128.7 ± 104.8	34.3 ± 15.8 ***
Labetalol E_{max} ratio	-0.07	0.03 ± 0.03	0.02 ± 0.03
Nicardipine E_{max}	-40.0	11.5 ± 13.2	6.3 ± 6.7 ***
Nicardipine EC_{50}	70.0	65.4 ± 107.2	11.0 ± 8.4 ***
Nicardipine E_{max} ratio	-0.19	0.05 ± 0.04	0.03 ± 0.03 ***

Mean and standard deviation of absolute errors from ground truth of estimated parameters from non-linear least squares (NLLS) and AIM-BP. Significantly lower AIM-BP absolute errors are noted with asterisks (*** = $p < 0.0005$, ** = $p < 0.005$, * = $p < 0.05$).
