# Supplementary figures and images for: Towards precision critical care management of blood pressure in hemorrhagic stroke patients using dynamic linear models
Source: PLoS One. 2019 Aug 5;14(8):e0220283. doi: 10.1371/journal.pone.0220283 (PMC6681940; doi:10.1371/journal.pone.0220283)

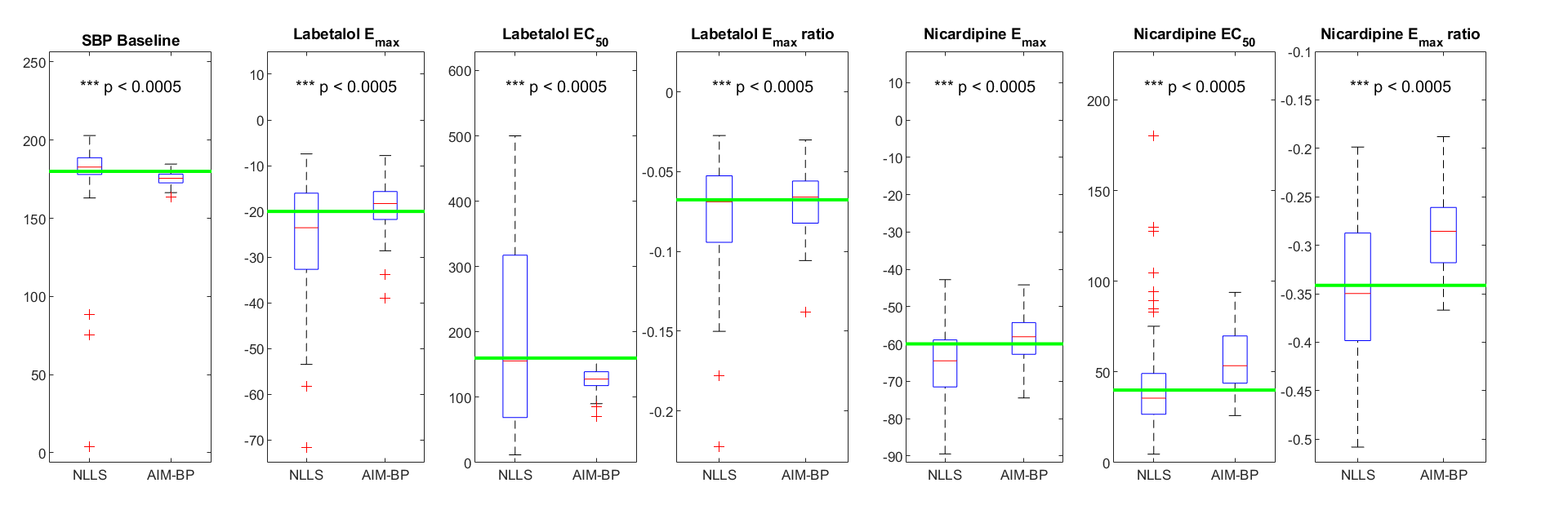

Supplement: S1 Fig — Box and whisker plots of estimated parameters using non-linear least squares (NLLS) and AIM-BP for Clinical Scenario 3. Significantly lower variances of AIM-BP estimated parameters compared to NLLS are noted with asterisks and p-values. (TIFF) [file pone.0220283.s005.tiff]

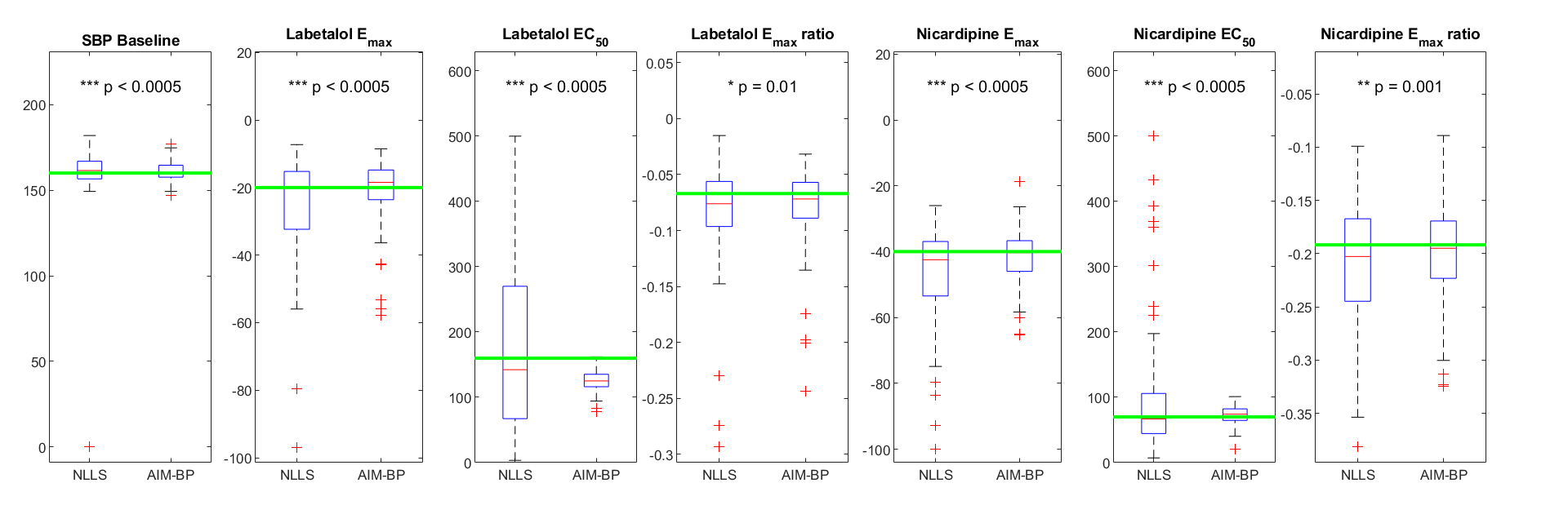

Supplement: S2 Fig — Box and whisker plots of estimated parameters using non-linear least squares (NLLS) and AIM-BP for Clinical Scenario 4. Significantly lower variances of AIM-BP estimated parameters compared to NLLS are noted with asterisks and p-values. (TIFF) [file pone.0220283.s006.tiff]

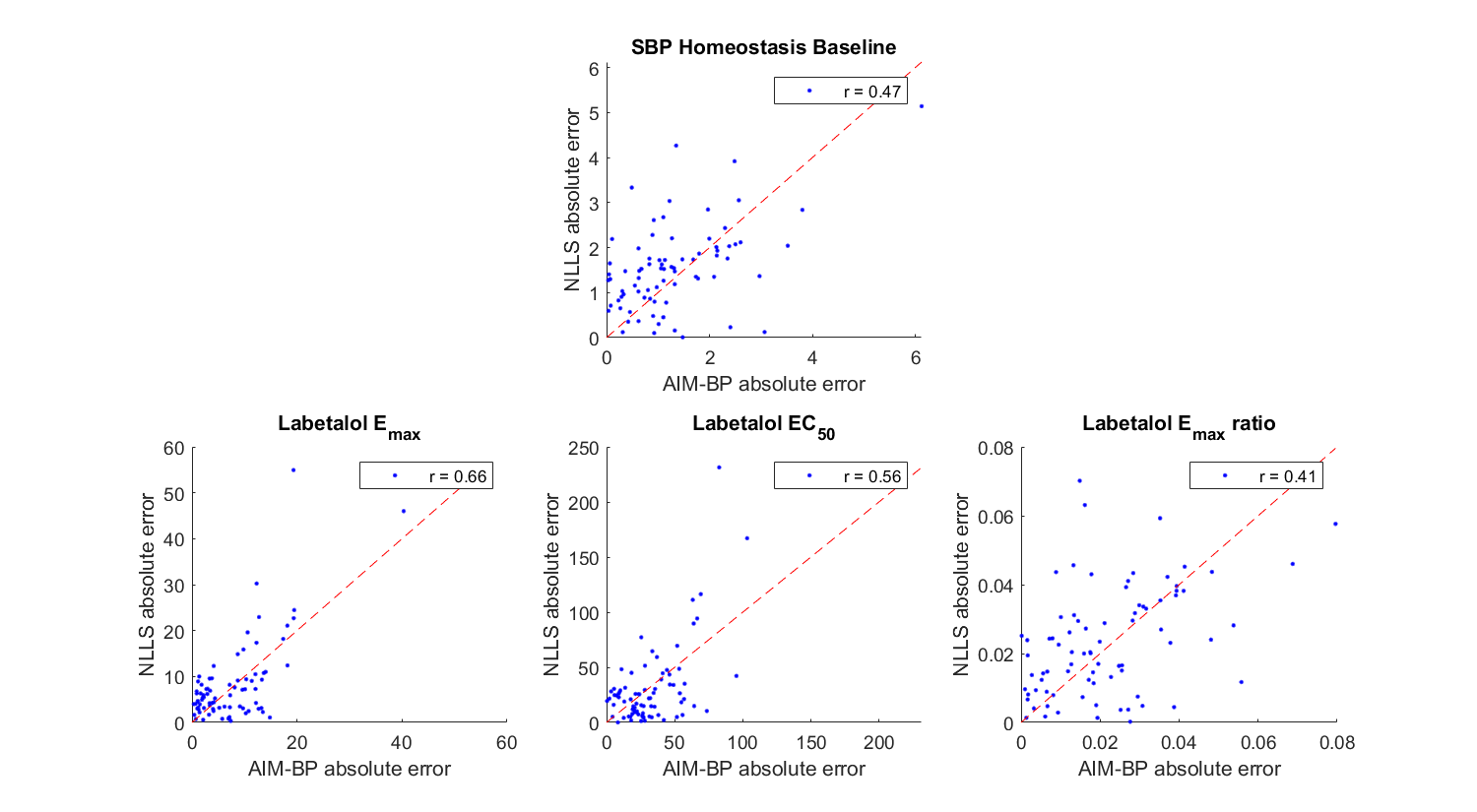

Supplement: S3 Fig — Absolute errors of SBP homeostasis baseline, Emax, EC50, and Emax ratio from AIM-BP estimates were plotted against absolute errors from NLLS estimates for Scenario 1. A Pearson correlation coefficient was calculated for each. (TIFF) [file pone.0220283.s007.tiff]

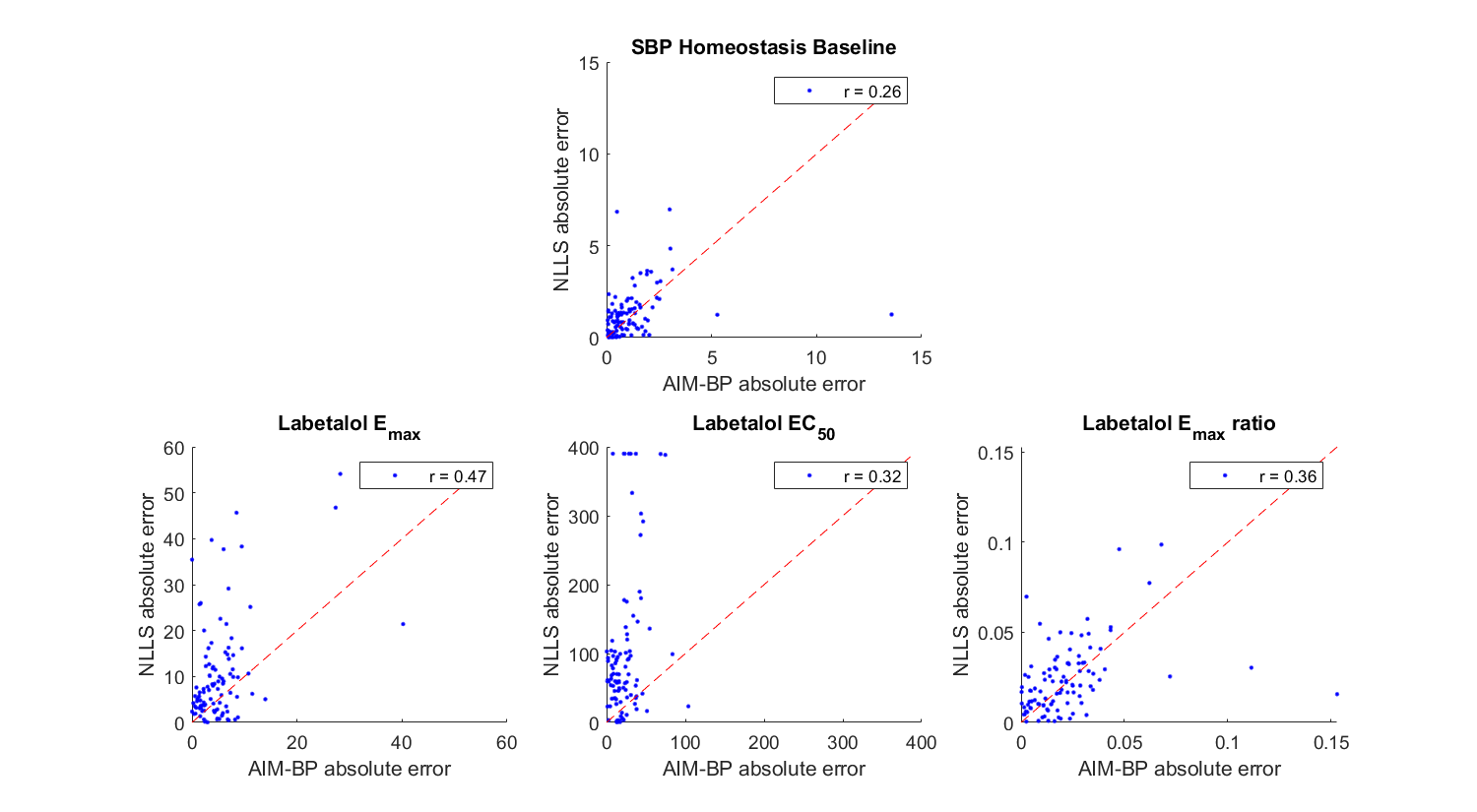

Supplement: S4 Fig — Absolute errors of SBP homeostasis baseline, Emax, EC50, and Emax ratio from AIM-BP estimates were plotted against absolute errors from NLLS estimates for Scenario 2. A Pearson correlation coefficient was calculated for each. (TIFF) [file pone.0220283.s008.tiff]

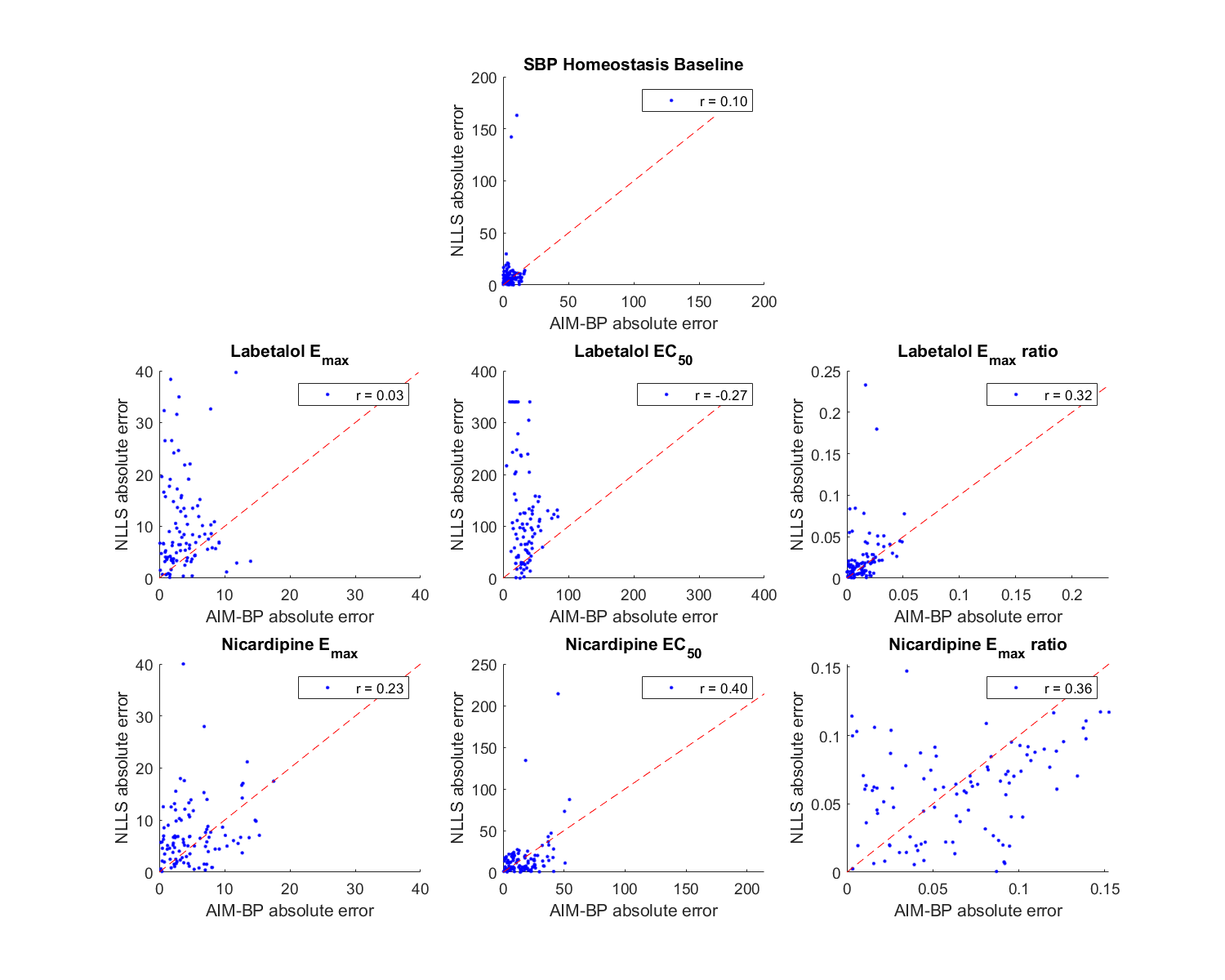

Supplement: S5 Fig — Absolute errors of SBP homeostasis baseline, Emax, EC50, and Emax ratio from AIM-BP estimates were plotted against absolute errors from NLLS estimates for Scenario 3. A Pearson correlation coefficient was calculated for each. (TIFF) [file pone.0220283.s009.tiff]

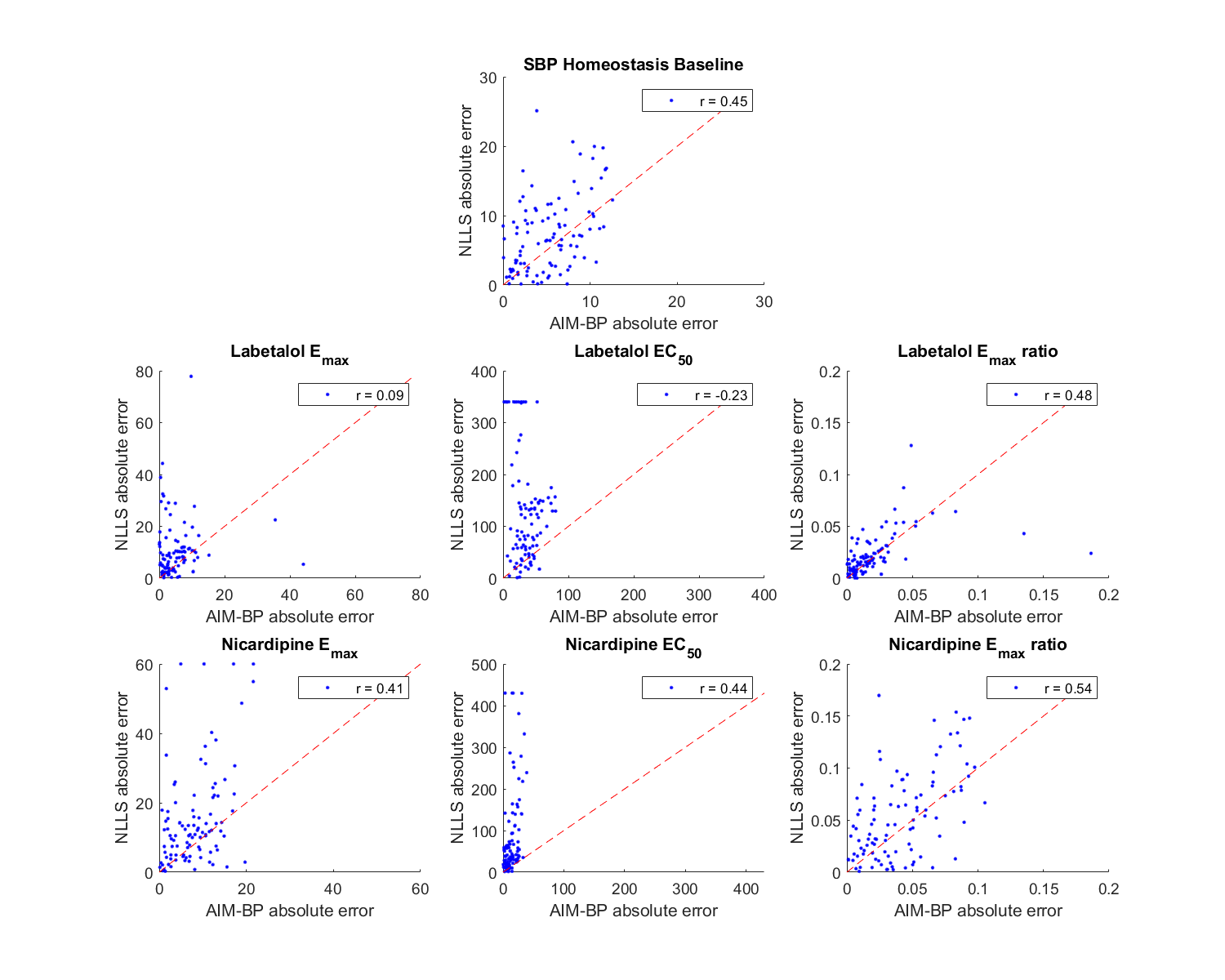

Supplement: S6 Fig — Absolute errors of SBP homeostasis baseline, Emax, EC50, and Emax ratio from AIM-BP estimates were plotted against absolute errors from NLLS estimates for Scenario 4. A Pearson correlation coefficient was calculated for each. (TIFF) [file pone.0220283.s010.tiff]
